# Design and testing of a RealSense-based variable spraying control system for field kale

**DOI:** 10.3389/fpls.2025.1618159

**Published:** 2025-08-04

**Authors:** Yahui Luo, Wen Li, Pin Jiang, Kaiwen Tang, Zhiluo Liang, Yixin Shi

**Affiliations:** College of Mechanical and Electrical Engineering, Hunan Agricultural University, Changsha, China

**Keywords:** pulse width modulation, variable spraying, improved YOLOv8n algorithm, atomization deposition density, binocular vision sensor

## Abstract

Precision PWM variable spray technology and target detection, identification, and localization technology are key to solving the pesticide waste associated with traditional constant application methods and to improving pesticide utilization for achieving precise application. To address the problems of high pesticide dosage, low application efficiency, and poor kale pest and disease control in traditional upland gap sprayers, a variable spray control system was designed in the study. The system utilizes binocular vision sensors to detect kale targets in the field in real time and achieves accurate pesticide application through pulse-width modulation technology. An improved target detection model based on YOLOv8n is presented, with experimental results showing a detection accuracy of up to 88.7% for field-grown kale. The system was also tested for accuracy-responsive variable spraying in recognition detection tasks, with a 0.2% reduction in the central atomized deposition density coefficient of variation (CV) compared to constant spraying. A flow on/off test model was designed for the solenoid valve duty cycle, determining the correlation decision coefficient for spraying. The correlation coefficient of the flow model exceeded 0.9958 when the duty cycle was in the range of 20–90%, and the actual and theoretical flow rates at the spray terminals were strongly linearly correlated, with a maximum error of only 4.1%. The spraying effect of the system was evaluated through field tests. The results show that the theoretical spray volume of the variable spray control system aligns well with the actual spray volume. In field atomization deposition tests, compared with constant-rate spraying, the target center atomization density in variable spraying mode reached 34.42%. Although droplet deposition and coverage around the crop were slightly reduced, pest and disease control around kale remained effective. In addition, the variable-rate spraying control system further improved pesticide utilization, with a maximum pesticide savings of 26.58%. This study demonstrates the feasibility of binocular vision sensor-guided spraying operations in field environments and provides a reference for its application in field pest control.

## 1 Introduction

Field kale is susceptible to soft rot, downy mildew, cabbage greenfly, aphids, and other pests and diseases during the growth process, which seriously affects its yield and quality, and poses a challenge to farmers’ economic returns and production stability (Zhao et al., 2019). As a cruciferous crop, kale has a short growth cycle and dense foliage, making it easy for pests and diseases to spread rapidly, and turning their control into a core aspect of field management (Wan et al., 2025). According to statistics, the kale growing period requires frequent pesticide spraying, with the number of applications in some areas reaching as high as 6 to 12 times per year, accounting for 25% to 40% of the total labor input for field management (Lan et al., 2022). Traditional chemical control mainly relies on backpack sprayers or manual spray bars for full-coverage spraying, where the liquid is dispersed into tiny droplets on the surface of the plant through high pressure (Jin et al., 2016; Li W. et al., 2023). However, this “uniform coverage, thorough wetting” model of rough application often ignores differences in canopy structure at different growth stages of kale, resulting in excessive spraying and associated environmental pollution (He, 2020). Studies have shown that under conventional continuous spraying conditions, the liquid has difficulty penetrating the inner leaf bulb due to leaf shading, resulting in overspray on the outer layer and underspray on the inner layer. Meanwhile, the runoff and drift ratio of the liquid can reach 30% to 50%, increasing production costs and risking soil contamination and pesticide residues (Zhai et al., 2018; Salcedo et al., 2020). During the precise pesticide application process, the hydrophobic nature of kale leaves poses a potential challenge that must be addressed. As the growing season progresses, the Leaf Area Index (LAI) of kale exhibits an upward trend, which is critical for adapting the spraying rate to match the plant’s developmental stage. Furthermore, in the early stages of growth, the lower kale leaves, which are closer to the ground, are particularly vulnerable to pest and disease infestations. Achieving comprehensive spray coverage, especially on these lower leaves, presents a significant difficulty that needs to be overcome. Addressing these issues is essential for optimizing the precision spraying system. Therefore, optimizing precision application strategies based on the dynamic growth characteristics of kale has become a key research direction to enhance pest control efficiency and promote sustainable agricultural development.

Variable-rate spraying technology is an intelligent application method that dynamically senses crop canopy parameters and adjusts the pesticide amount in real time (Han et al., 2024). As a precision plant protection technology, it can significantly reduce pesticide waste and environmental pollution in kale cultivation under the traditional continuous spraying model through differentiated application (Zhang et al., 2017). Current research on variable-rate spraying focuses on two main areas: (1) canopy feature recognition using multi-source sensors, including real-time monitoring of kale plant density, leaf bulb maturity, and canopy volume index through LiDAR, spectral sensors, or machine vision systems (Jiang et al., 2019; Shu et al., 2020; Xue et al., 2022); and (2) execution control systems that calculate target application rates based on pest and disease levels and canopy characteristics, using PWM to achieve variable spraying (Dai et al., 2019; Fan et al., 2021). This approach enables precise output for different flow rates and adaptive droplet size adjustment, optimizing coverage of the outer leaves and penetration into inner layers (Zhao et al., 2022). Current technical bottlenecks include limited sensor feature extraction accuracy in dense leaf bulb environments, and challenges in dynamically matching application systems to agricultural machinery speed, which require further breakthroughs to achieve fully intelligent kale spraying (Wang et al., 2022).

The core of variable-rate application technology lies in the precise acquisition of crop canopy characteristics. Current target detection technologies mainly integrate machine vision, laser sensors, ultrasonic sensors, radar localization sensors, and multi-sensor fusion (Yuan et al., 2020; Gu et al., 2021; Dou et al., 2022; Yang et al., 2022). Laser sensors, though capable, are hindered by their sensitivity to humidity and dust in the kale field environment (Yan et al., 2021). While LiDAR can reconstruct canopy structure via high-precision point clouds, it is poorly suited to field conditions due to low adaptability to dynamic scenes, complex data processing, and high power consumption (Wang et al., 2023). Ultrasonic sensors, which detect distance via acoustic time-of-flight, show canopy volume measurement errors of 12%–18% during kale nodulation due to uneven leaf bulb surfaces causing multipath reflections (Zhai et al., 2022). Although they have a longer detection range than LiDAR, their slower response time makes them unsuitable for real-time detection (Luo et al., 2024). In contrast, machine vision can achieve over 90% plant recognition accuracy under the same conditions through sub-pixel edge detection and millisecond-level image processing, making it more adaptable to high-density, dynamic field applications (Tewari et al., 2020; Wang G. et al., 2024). The Intel RealSense family of binocular vision sensors combines infrared-assisted and laser-assisted modules, enabling accurate volume detection of kale leaf bulbs and real-time monitoring of crop growth. This integration of infrared, laser, and machine vision technologies enhances its performance in complex field environments (Zhao et al., 2022; Qiao et al., 2024; Xu et al., 2024).

In variable-rate spraying systems, fusing canopy feature sensing with dynamic flow regulation models provides an efficient solution for precise pest and disease control in kale (Li Y. et al., 2023). A decision coefficient model based on leaf wall area and canopy density has been shown to quantify the impact of the laminated leaf bulb structure on spray penetration during the nodulation stage (Xue et al., 2020). By optimizing the multi-nozzle synergistic flow function, pesticide utilization was increased to 68.34% in field trials, significantly reducing leaf-core liquid stagnation risk (Hussain et al., 2020). In low-frequency regulation, spray flow is primarily governed by the duty cycle, with pressure contributing over 70% to flow fluctuations (Zhai et al., 2022). In high-frequency settings, flow ranges from 50–500 mL/min, though the linear response interval narrows to a 40%–60% duty cycle, necessitating a pressure compensation PID algorithm to suppress pulsation effects (Wang Z. et al., 2024). Dynamic kale growth characteristics are further captured by identifying leaf bulb maturity via multispectral imaging, distinguishing the outer loose leaf layer from the inner dense bulb (Han et al., 2024). High humidity and dusty field conditions further complicate pest management, but are addressed through pressure-duty cycle decoupling control and multi-nozzle coordination strategies (Han et al., 2024). Introducing a feed-forward-feedback composite controller reduced flow deviation due to pressure fluctuations from 12% to under 3% (Zhu et al., 2010).

To improve the dynamic adaptability of field kale application units to canopy volume and spraying characteristics, this study focuses on the synergistic optimization of target detection and variable-rate regulation technologies. The detection system, based on the RealSense binocular vision sensor, and the theoretical control method for variable-rate spraying, provide both technical and theoretical support for precision protection of high-density canopy crops. Through field trials, the study analyzes the duty cycle–flow decision model and canopy parameter–application volume decision model, comparing constant-rate spraying with canopy volume index–based spraying to evaluate fog deposition effects and provide a theoretical basis for pest control and precise application under high humidity.

## 2 Materials and methods

### 2.1 Variable rate spray system

**Figure 1:**
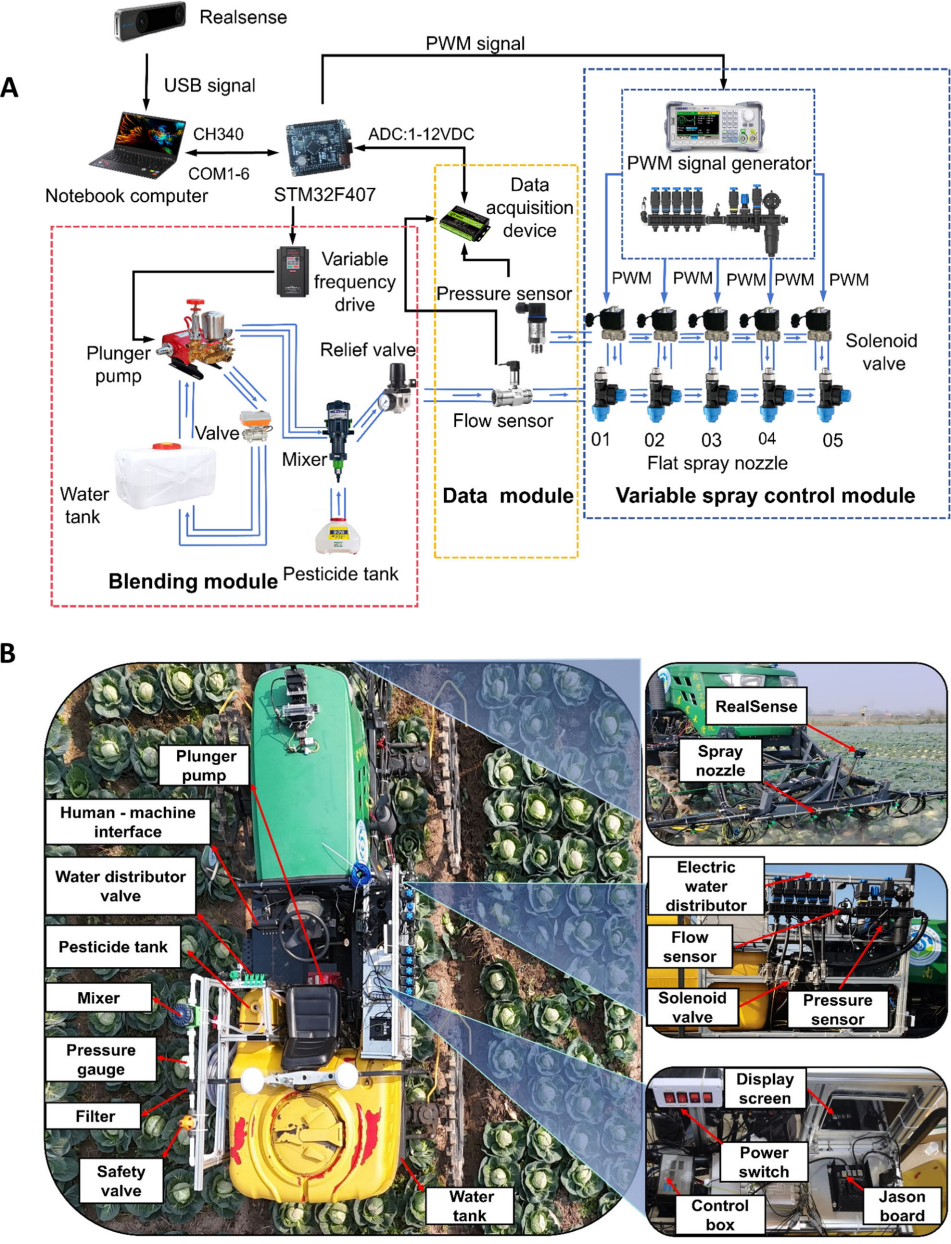
Variable rate spraying system: **(A)** Basic components of the system **(B)** Overall structure.

In open - field kale cultivation, pests and diseases such as soft rot, downy mildew and caterpillars are common threats. The pesticide selection for spraying is determined by the actual pest/disease situation and control requirements to fit the growth process of kale. In the constructed variable - rate spraying system, azoxystrobin and chlorbenzuron, typical pesticides, are primarily used. Meanwhile, in the experiment, water and carmine are adopted as substitutes to ensure operator safety. The architecture of the variable-rate spraying system developed in this study is shown in **Figure 1A**. The system comprises a mixing unit, data acquisition unit, variable-rate spraying unit, and target detection unit. The target detection unit uses the RealSense D455 binocular vision sensor to recognize kale canopy volume and bullseye position in real time. The visual module, with an improved YOLOv8n algorithm, enables accurate kale recognition and location in the variable - rate spraying system. The system integrates PWM technology to control the electromagnetic valve at different duty cycles, thereby adjusting spray parameters and ensuring precise pesticide application on kale. Collected data are transmitted to an embedded controller via CAN bus; the PWM control command is generated by the application decision model and sent via RS-485 protocol to the variable-rate control module, which activates the 2KS200 solenoid valve array for dynamic spraying. A PC interface displays real-time data on plant density and application rates, achieving closed-loop “perception–decision–execution” control. The STM32F407 microcontroller (STMicroelectronics) serves as the lower computer, receiving and processing upper computer commands via RS-485 protocol. The hardware includes a spray mixing unit (with a plunger pump, Mixtron volumetric dispenser with 0.2% accuracy, and electric ball valve), a data acquisition unit (RealSense D455 sensor, ± 2 mm depth accuracy), and a spraying unit (2KS200 solenoid valves, W25–04 flat-fan nozzles with 110° atomization angle and 0.4–1.2 L/min flow rate, ZK five-way motorized valves). The W25–04 in the system is a standard flat - fan nozzle with anti - leakage function. The system is mounted on a high-clearance sprayer (1.3 m above ground) with a modular left–center–right structure spaced at 60 cm. The system’s physical layout is shown in **Figure 1B**.

**Figure 2:**
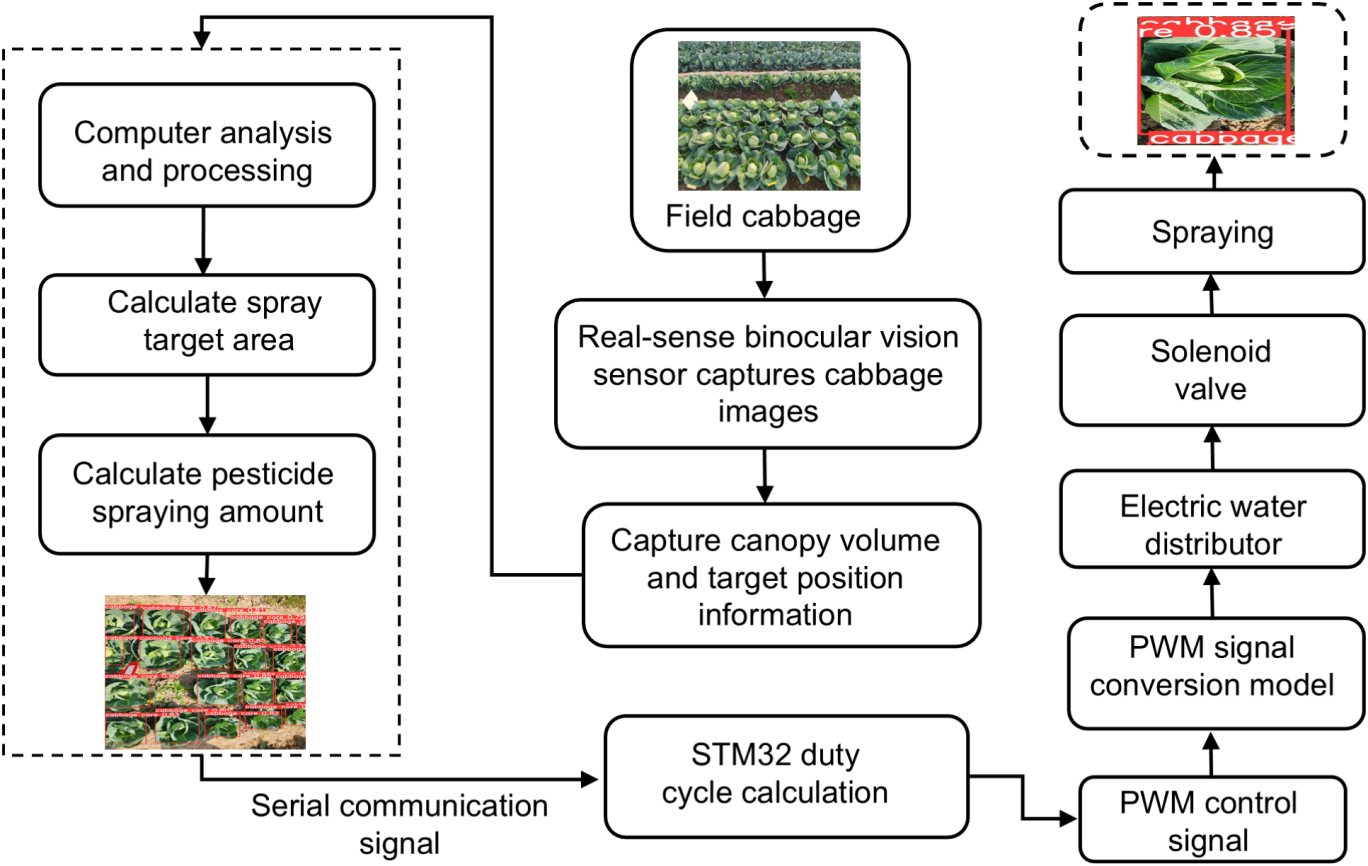
Variable rate spray system control flowchart.

Within the variable-rate spraying system, the water and pesticide tanks are separate. The plunger pump and Mixtron volumetric dispenser draw water and pesticide respectively, mixing them accurately to maintain a stable concentration. The mixed pesticide circulates through a loop involving these components and valves in the mixing unit, then flows to the pressure and flow sensors in the data acquisition module. Finally, it is transported via pressure-resistant pipes to the variable spraying module, where electromagnetic valves regulate the spray from the fan-shaped nozzles. The overall control principle and process of the variable spraying system are shown in **Figure 2**. The system captures images of the volume and bullseye position of the kale canopy in the field using binocular vision sensing. These images are transmitted to a PC for processing to determine the size of the leaf bulb volume, after which the application dosage decision-making model calculates the target spraying volume. The core control unit converts this target volume into a duty cycle, which is then used as dynamic duty cycle information for targeted spraying. This information is transmitted via serial communication to the STM32 microcontroller, which outputs PWM signals to drive the solenoid valve and simultaneously control the motorized five-way water dispensing valve to execute the spraying commands.

### 2.2 Variable rate spray system identification model construction

#### 2.2.1 Improvement of YOLOv8n algorithm

Kale, as a typical canopy-intensive crop, is an important cruciferous vegetable whose agronomic planting parameters are closely tied to the requirements of precision application technology. According to ISHS standards and actual production needs, the recommended planting spacing for kale is 40–60 cm. This spacing not only accommodates nodulation but also allows sufficient space for full leafball expansion during that stage, helping to prevent poor ventilation, reduced light transmission, and disease resulting from overly dense planting. Regarding application rates, due to the thick waxy layer of kale leaves and its dense canopy, the recommended spraying volume is 400–600 L/ha, which is 25%–33% higher than that of leafy vegetables such as cabbage (Zhang et al., 2017).

**Figure 3:**
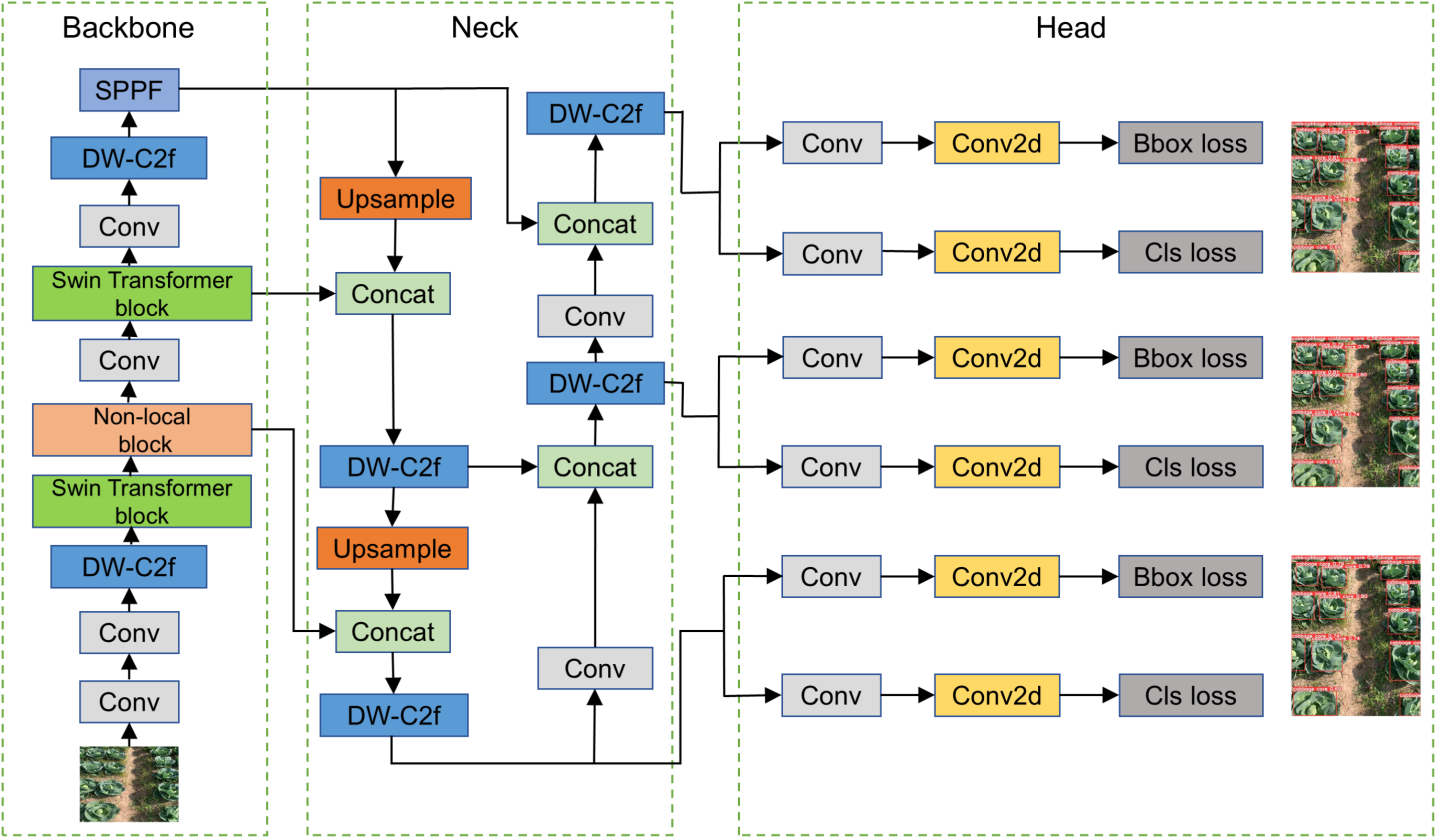
General architecture of the YOLOvn8 kale recognition model.

In this study, kale was cultivated according to standard agronomic practices with a plant spacing of 50 cm and a row spacing of 60 cm. The current YOLOv8 algorithm is widely adopted for high-density crop target detection due to its high efficiency, accuracy, and reliability, making it a popular choice in vegetable recognition applications. The complexity of kale field detection scenarios—such as small target scale and strict real-time requirements—is systematically evaluated. A comparative analysis was conducted on the migration learning performance of five commonly used models—Faster R-CNN, YOLOv8n, SSD, YOLOv5S, and YOLOv8—to determine the most suitable detection model for kale (Fu et al., 2022). While Faster R-CNN, a two-stage detection framework, excels in small target detection in complex scenes by generating candidate regions for feature extraction and classification, its time-consuming process and high deployment costs make it unsuitable for real-time kale detection. SSD, with single-stage detection and multi-scale feature mapping, underperforms on small targets due to insufficient shallow feature resolution. YOLO’s unique grid-based architecture enables efficient multi-category detection with fewer computational resources. YOLOv8 replaces the C3 structure in YOLOv5 with the C2f structure for improved gradient flow and modifies channel numbers for different model scales. As a result, even lightweight models like YOLOv8n perform well, particularly for lower-resolution images captured by mobile devices. In testing, YOLOv8n demonstrated the fastest detection speed and best performance among the lightweight models. Accordingly, YOLOv8n was selected in this study and further optimized to construct a recognition model capable of fast, accurate detection of field-grown kale under high-density, high-humidity conditions. Its architecture is illustrated in **Figure 3**.

The constructed kale recognition model consists of three main parts: backbone, neck, and head. The backbone is responsible for extracting image feature information of kale and feeding it into the subsequent network. The Conv module contains convolutional layers, batch normalization, and activation functions for standard feature extraction. The DW-C2f module performs feature extraction and fusion using connections across different bottleneck layers. The Swin module enhances detection accuracy in edge regions of the kale image, thereby improving overall recognition precision for better variable spraying. The SPPF module fuses features that have undergone a maximum of three max-pooling operations. This sequence enables efficient extraction and fusion of rich feature information. The neck connects the backbone and head and facilitates deeper feature fusion. It integrates multiple Concat and DW-C2f modules to combine low- and high-level features after several convolution operations. The left and right sides of the head perform object detection in conjunction with binocular vision sensors, with a Bbox loss module for bounding box regression and a Cls module for classification.

The preliminary YOLOv8n detection model, while capable of identifying target crops in the field, exhibits inadequate adaptation to dense kale plantations. To improve this, the study uses larger convolutional kernels and depthwise convolutions to lower computational costs (Chen et al., 2024). Depthwise convolution decreases floating-point operations and parameters by applying each kernel to a single input image channel. Although dilated convolution can expand the receptive field, it might impact downstream tasks. To address this, the study employs a dilated convolution design with added shape biases to enhance model generalization and reduce overfitting risks (Ma et al., 2018). ConvFFN modules replace the original ones in the bottleneck to expand the model’s receptive field and improve feature extraction for target kale. The original YOLOv8n struggles with detecting edge regions in kale images due to insufficient convolutional operations and weak gradient updates for edge pixels. To solve this, the study integrates a Swin Transformer module with CNN. The Swin Transformer’s self-attention mechanism better extracts edge semantic information, overcoming CNN’s edge detection limitations and improving target semantic understanding (Nevavuori et al., 2019). In field scenes, kale is often occluded by weeds and other objects. A non-local attention mechanism is added to the algorithm to enhance feature extraction of kale in practical detection and strengthen the model’s ability to capture long-range dependencies. Channel or spatial attention mechanisms aim to generate more discriminative features and improve the distinctiveness of key features (Thenmozhi and Srinivasulu Reddy, 2019). However, channel-wise dimensionality reduction may affect the visual representation of target kale. Therefore, a spatial domain attention method is used. It transforms spatial information from the original image to another space while retaining key details, avoiding the adverse effects of channel attention mechanisms and improving target identification in high-density field scenarios.

The performance of five mainstream detection models—Faster R-CNN, YOLOv8n, SSD, YOLOv5S, and YOLOv8—was tested on a kale dataset collected from a large field. The hardware platform for the test was the NVIDIA Jetson AGX Orin (32 GB video memory, CUDA 11.4), used to comprehensively evaluate the recognition accuracy of the models. Evaluation parameters such as mean average precision (mAP), image processing time, precision (P), and recall (R) (Wu et al., 2024) were adopted as the four criteria for model evaluation.

### 2.3 Kale target center canopy spray identification model construction

**Figure 4:**
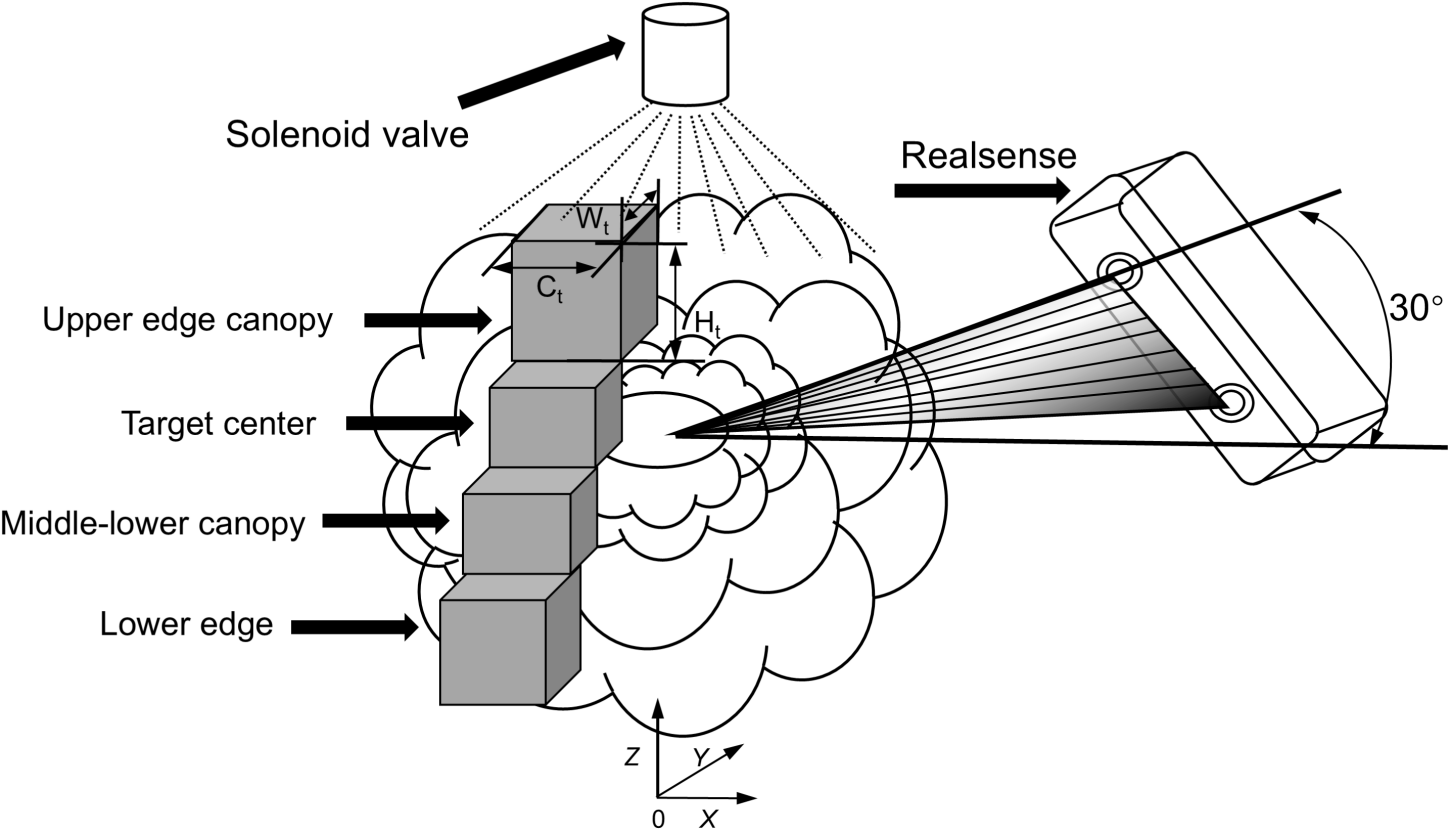
Schematic of the target center coronal density-volume spray identification model.


The kale planting parameters in the experimental field were a plant spacing of 60 cm, a row spacing of 50 cm, and a ridge spacing of 1.2 m, which meet agronomic specifications for vegetable cultivation. Based on field planting density and the camera’s imaging recognition principle, the RealSense sensor was mounted at a height of 80 cm, with a horizontal offset between the spray bar’s central axis and the sensor set at 25 cm. The sensor’s installation position can be adjusted to adapt to various planting modes, maximizing target spraying accuracy. In the study, a canopy density–volume spray identification model was constructed based solely on field kale agronomy, as shown in **Figure 4**. The computational method is also illustrated. The model computes RGB and depth data from kale canopy images in real time using the binocular vision sensor. The derived model triggers the solenoid valve for spraying, while the actual height and width of the detected target are calculated using the imaging principle (Qi et al., 2023), as shown in Equation 1.

(1) $$\frac{\text{f}}{\;\text{R}/2-\text{e}}=\frac{\text{Hp}}{\text{Ht}}=\frac{\text{Wp}}{\text{Wt}}$$

where f is the sensor focal length, in this paper, f = 3.95 mm; H_p_ the pixel height of the measurement area in mm; H_t_ is the actual height of the detection area in mm; W_p_ is the pixel width of the detection area in mm; W_t_ is the actual width of the detection area in mm.

In field conditions, the tight layering of kale leaves during the nodulation stage often causes local occlusion, increasing the error rate in binocular vision feature point matching and affecting recognition accuracy. To improve pest control effectiveness, targeted spraying was enhanced by reconstructing effective pesticide application regions. Based on morphological characteristics—plant height 30–50 cm, consistent with NY/T 1837–2010 planting standards—the bullseye position of the kale canopy was selected as the spraying target. To address canopy occlusion and shading, the canopy was segmented into upper edge, bullseye region, center, and lower edge canopies (Li et al., 2018). Given kale’s generally low stature, the binocular sensor’s pitch angle was set at 30°, and its resolution was configured to 240×320 pixels.

To systematically evaluate the field adaptability of the improved spray recognition model, several rows of kale were selected for experimental testing. Results were compared to the baseline YOLOv8 model to assess algorithmic improvements (Hussain et al., 2020). During the test, the system moved at a constant speed of V = 1 m/s, with kale canopy width W_target_​ collected in real time via the integrated binocular vision sensor. Pre-processed target feature parameters and a fixed detection distance were input to the model. The variable-rate spraying system transmitted the detected kale data to the core controller, which dynamically adjusted solenoid valve timing using the response frequency model derived in Equations 2-4. In addition, rigid coupling between the binocular sensor and solenoid valve array at the front end of the folding spray bar minimized positional error from vibration, ensuring spatial synchronization between target detection and spray execution, and significantly improving spraying accuracy.


(2) $${\text{T}}_{1}=\frac{{\text{W}}_{\text{Target}}}{\text{V}}$$

(3) $${\text{T}}_{2}=\frac{\text{d}}{\text{v}}$$

(4) $${\text{f}}_{1}=\frac{1}{{\text{T}}_{1}+{\text{T}}_{2}}$$

where W_Target_ is the width of the target kale in cm; d is the detection distance corresponding to the target crop in cm; v is the forward speed of the machine in m/s; f_1_ is the switching frequency of the solenoid valve in Hz; T_1_ is the opening time of the solenoid valve in s; and T_2_ is the closing time of the solenoid valve in s.

### 2.4 PWM duty cycle-flow rate decision modeling

#### 2.4.1 System spray flow rate accuracy test

The response speed of the solenoid valve in the variable-rate spraying system directly influences flow control precision. In pest control operations for field kale, pesticide spray parameters must be dynamically adjusted based on crop growth and pest types. Because different pesticides cover different areas, constructing a theoretical mathematical flow decision model is crucial for achieving precise system-level flow control. The theoretical model for application rate per unit area in high-density kale fields (Zhang H. et al., 2024) is given in Equation 5.


(5) $${\text{Q}}_{\text{plant}}=\frac{{\text{Q}}_{\text{area}}\cdot \text{C}}{{\text{N}}_{\text{plant}}}$$

where Q_area_ for the unit area of the amount of drug application, the unit is L/ha; C for the active ingredient concentration of the agent, the unit is %; N_plant_ unit area planting density, the unit is plant/ha.

There are significant differences in the canopy density of kale, and its canopy density and volume parameters directly affect the opening and closing control logic of the solenoid valve. Canopy density of kale refers to the number and distribution of kale leaves per unit area, serving as a vital measure of plant vigor and leaf coverage in the field. In the dynamic spraying management system for different kale canopy areas, a stereo visual sensor first captures the canopy’s volume and center position. The core processor then estimates pest and disease risks and analyzes the canopy density for each area. For the upper canopy edge, where less pest and disease occur due to more light, the system reduces the duty cycle via PWM signals to decrease pesticide application. The center area, with dense leaves and high pest/disease risk, is managed by the system module, which dynamically adjusts the electromagnetic valve’s duty cycle based on YOLOv8n’s visual feedback. This optimizes liquid distribution for precise spraying. For the lower canopy edge, which is near the ground and hard to spray, the system increases spray pressure using image data to enhance droplet penetration, ensuring effective coverage. In general, the higher the canopy density, the greater the corresponding required pesticide dosage. A three-dimensional canopy volume characterization was constructed based on canopy area and density (Manandhar et al., 2020). In this study, a pesticide flow decision model, Kcv, centered on canopy density is established for verifying the dynamic response characteristics of the PWM duty cycle flow model, and the model expression is shown in Equation 6.


(6) $$\text{Kcv}=0.5\times \frac{\text{NLWA}}{\text{NALLI}}+0.5\times \frac{\text{Cw}}{\text{Cmax}}$$

where C_max_ is the maximum coverage area per unit area of the spray target in m^2^; NLWA is the normalized leaf weighted area in m^2^; NALLI is the normalized leaf anisotropic blade index; C_W_ pesticide maximum threshold.

Further, the PWM duty cycle–flow theoretical model Q(D, P) (Wen et al., 2018), whose expression is shown in Equation 7, is defined and embedded into the control unit of the variable-rate spraying system, controlled by adjusting the duty cycle of the solenoid valves’ opening and closing in real time.


(7) $$\text{Q}=\{\begin{array}{c} \;\;\;\;0\;\;\;\;\;\;\;\;\;\;\;\;\;\;\;\;\;\;\;\;\;\;\;\;\;\;\;\;\;\text{D}\leq 15\%\\ \text{k}{\text{P}}^{n}{\left(\frac{\text{D}-15}{70}\right)}^{1.32}\;\;\;\;\;\;\;\;\;\;\;\;\;\;\;\;\;\;\;\;\;15\%<\text{D}<85\%\\ \;\;\;\text{k}{\text{P}}^{n}\;\;\;\;\;\;\;\;\;\;\;\;\;\;\;\;\;\;\;\;\;\;\;\;\;\;\;\text{D}\geq 85\% \end{array}$$

where K = 0.694 is the flow coefficient, n = 0.583 is the pressure correction index, in line with ISO 16119–3 standard verification.

To verify the theoretical flow rate of the model and the control accuracy of the actual spraying unit, a spraying flow rate test was conducted to evaluate the opening and closing response characteristics of the solenoid valve under synergistic regulation with the PWM duty cycle signal. The test included four pressure gradients: 0.1 MPa, 0.2 MPa, 0.3 MPa, and 0.4 MPa, and duty cycle tests ranging from 10% to 90% under a 10 Hz control signal. Preliminary tests showed that within the low duty cycle range of 20%–40%, due to the imbalance in solenoid valve opening and closing time, there were significant fluctuations in flow output, and the terminal effective spray volume was insufficient to meet the pest prevention threshold requirements. In the 60%–80% duty cycle range, the system flow stability significantly improved, and pipeline pressure fluctuations were minimal. To quantify the control characteristics, the test was repeated using a flowmeter across 5 duty cycle gradient groups, each group maintained for 30 s for system stabilization. Data were collected and averaged over 5 repetitions to determine the effective output value.

#### 2.4.2 Crop canopy spray flow accuracy tests

To evaluate the variable control accuracy of the variable-rate spraying system in a field kale environment, a graded control experiment was designed, including a dynamically adjusted variable spraying group and a fixed-flow constant spraying group for comparison. Based on the morphological characteristics of the kale canopy (plant height 30–50 cm, row spacing 50 cm) (Wang Z. et al., 2024), the experimental target area was divided into three parts: the upper canopy edge (0–10 cm from the top), the target center (agronomic core control area), and the lower canopy edge (0–10 cm from the base). Flow data for each area were collected in real time using a Coriolis mass flowmeter (accuracy ±0.5%). During the test, the sprayer traveled at a constant speed of 1 m/s, the system working pressure was set to 0.3 MPa, and each test group was repeated three times with averaged results to reduce random error. For safety, water was used instead of pesticides, and line pressure stability was maintained using a pressure compensation valve. Different spraying methods were used to evaluate flow accuracy across kale target areas.

### 2.5 Field test

#### 2.5.1 Verification test of atomized deposition performance

**Figure 5:**
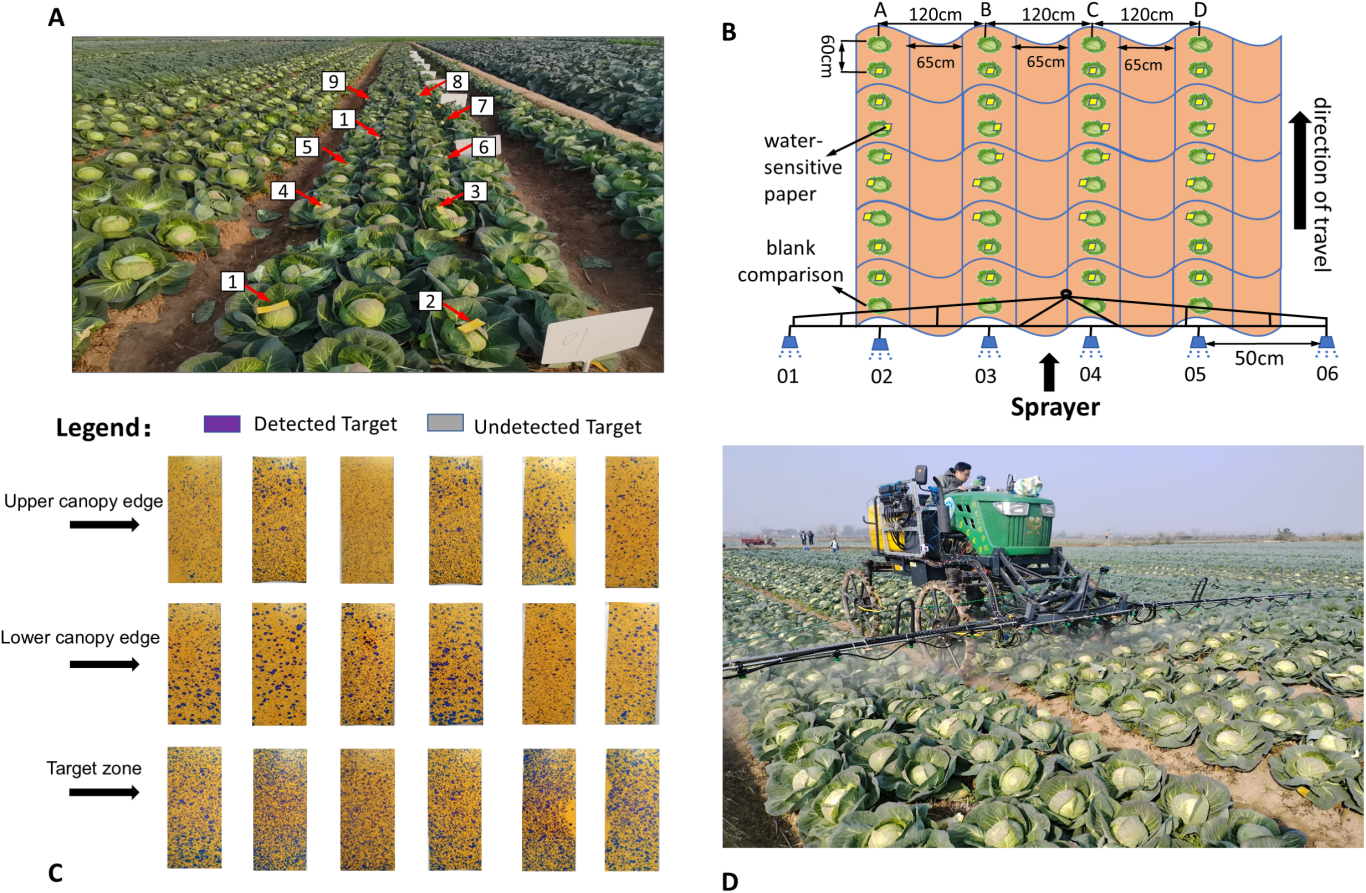
Field experiment tests: **(A)** Water-sensitive paper sampling point layout **(B)** Schematic layout of atomized deposition **(C)** Water-sensitive paper after field experiment tests **(D)**Field test experiment site.


In agricultural plant protection operations, droplet deposition performance directly determines pesticide utilization and pest control efficiency. In this study, fog droplet deposition density, deposition amount, and coverage were used as core evaluation indicators. Field performance validation of the RealSense D455 binocular vision-based variable rate spray system was conducted. The experiment took place on December 3, 2024, at a vegetable plantation site in Junshan District, Yueyang City, Hunan Province (29.22°N, 112.88°E), under sunny, windless conditions with a temperature of 13.8 ± 0.5°C and relative humidity of 36%–43%. A control group (constant flow rate of 2.0 L/min) and a CV dynamic regulation treatment group were tested. Sampling points were arranged according to ISO 24253-2:2015 Plant Protection Machinery - Test for Arable Crops Deposit, as shown in the schematic diagram in **Figure 5B**. In the figure, each ridge of kale comprises 4 rows. In **Figure 5B**, A/B/C/D correspond to the respective rows of kale. Notably, the kale is planted in accordance with standard agronomic practices: a ridge spacing of 1.2 m, row spacing of 65 cm, and plant spacing of 60 cm. Based on the morphological characteristics of field kale, the target area was divided vertically into three gradients: upper canopy edge, target center, and lower canopy edge. For each kale group, 5–10 pieces of water-sensitive paper were placed in the same area, and cochineal solution was used in place of pesticide. Droplet coverage $C_{d}$ (%), deposition density $\rho_{d}$ (drops/cm^2^), and deposition per unit area V_d_ (uL/cm^2^) (Salcedo et al., 2021) were calculated according to Equations 8-10.

(8) $$\;\;{\text{C}}_{\text{d}}=\frac{{\text{A}}_{1}}{{\text{A}}_{2}}\times 100\%$$

(9) $${\text{ρ}}_{\text{d}}=\frac{{\text{N}}_{\text{d}}}{{\text{A}}_{2}}$$

where A_1_ indicates the droplet coverage area of the water-sensitive paper in cm^2^; A_2_ indicates the total area of the water-sensitive paper region in cm^2^; $\rho_{d}$ is the droplet deposition density in drops/cm^2^; N_d_ indicates the total number of droplets in the water-sensitive paper region, which is automatically counted by the image analysis software.


(10) $${\text{V}}_{\text{d}}=\frac{\text{C}{\text{e}}_{1}}{\text{C}{\text{e}}_{2}}\times \frac{\text{V}}{\text{S}}\;\times 10^{3}$$

where Ce_1_ indicates the percentage of cochineal in the eluent, i.e., the corresponding concentration; V is the volume of eluent in mL; Ce_2_ is the concentration of cochineal in the spray stock in ml/cm^2^; S indicates the area of water-sensitive paper in cm^2^.

Water-sensitive paper (Chongqing Liuliushanxia Plant Protection Science and Technology Co., Ltd.) and cochineal reagent (Fuzhou FeiJing Plant Science and Technology Co., Ltd.) were used. The paper size was 110 mm × 35 mm, with an effective detection area of 385 cm^2^. A 0.5 g/L cochineal solution was prepared with pure drinking water and detected at a wavelength of 507 nm using a Shimadzu UV-1240mini spectrophotometer, in accordance with GB 12475-2006. The kale planting parameters followed a monopoly planting pattern: four rows per monopoly, row spacing 65 cm, plant spacing 60 cm, monopoly width 1.2 m, in line with DB62/T 1978-2021. Water-sensitive paper was placed in three canopy zones: target center (stem–leaf junction), upper edge (0–15 cm from top), and lower edge (10–20 cm from ground), with ten sampling points per area, spaced at 60 cm in **Figure 5A**. A blank control group was included in a non-sprayed area. Spray pressure was 0.3 MPa, the sprayer moved in a straight line along the ridge at 1 m/s (**Figure 5D**), and water-sensitive paper was deployed 30 min prior to spraying to prevent environmental humidity interference. The variable-rate group adjusted flow in real time (1.5–3.0 L/min) using RealSense D455 data; the constant-rate group maintained 2.5 L/min at 0.3 MPa. Water-sensitive paper was recovered 5 min post-spray, dried in light, then scanned and analyzed using DepositScan software (Tewari et al., 2020). Each test group was repeated three times, outliers removed, and average values taken. Coverage and deposition density were calculated using Equations 8 and 9.

#### 2.5.2 Calibration of droplet deposition and uniformity evaluation

To quantify droplet deposition on kale canopy, water-sensitive paper from the test (**Figure 5C**) was analyzed. To guarantee the safety of operators, carmine solution (which is made of biological reagents) was used in the experiment instead of pesticides. The cochineal solution was calibrated via UV spectrophotometry with the UV-1240mini, and eluent absorbance (ABS) was measured. A linear regression equation was established (Equation 11) through standard concentration gradient testing. Droplet spatial distribution uniformity and liquid solution permeability were found to be kinetically coupled in agricultural spray deposition. When conducting field operations, the spray droplets landing on the water-sensitive papers placed in the kale field cause a physical diffusion phenomenon. To prevent the liquid solution’s physical diffusion from affecting the spray deposition results, the water-sensitive papers should be photographed promptly during the experiment. Based on plant surface interface science, the coefficient of variation (Dai et al., 2019) was used to evaluate uniformity (Equations 12-14), calculated as follows:


(11) $$\text{ABS}=24.381{\text{C}}_{\text{e}}+0.002({\text{R}}^{2}=0.998)$$

where ABS is the absorbance value of the measured solution; C_e_ is the concentration of the measured cochineal solution in g/L.


(12) $$\text{CV}=\frac{\text{δ}}{\text{μ}}\times 100\%$$

Format:


(13) $$\text{u}=\frac{\sum_{\text{i}=1}^{\text{n}}{\text{u}}_{\text{i}}}{\text{n}}$$

(14) $$\text{δ}=\sqrt{\frac{\sum_{\text{i}=1}^{\text{n}}{\left({\text{μ}}_{\text{i}}-\text{μ}\right)}^{2}}{\text{n}-1}}$$

where CV is the coefficient of variation; δ is the sample standard deviation; u is the sample mean; u_i_ is the sample observation; and n is the number of samples in the data set.

#### 2.5.3 Validation of solenoid valve dynamic response and variable spraying performance

To validate the target flow accuracy and dynamic response of a binocular vision-guided variable-rate spraying system, this study focused on the coupling between solenoid valve switching frequency and spray volume. Operation efficiency was optimized by adjusting sprayer speed. Experiments were conducted per JB/T 9782–2014 Test Methods for Plant Protection Machinery (Wang et al., 2023). Based on field agronomy and anticipated variable spray response delay, solenoid valve flow at different start/stop frequencies was tested to identify discrepancies between actual and theoretical spray volumes. Sprayer speed was adjusted across four groups: 0.5 m/s (low), 1 m/s (benchmark), 1.5 m/s (medium), and 2 m/s (high), tested with different duty cycles. Spray volume during a single valve cycle and over 1 min was measured using a standard 1 mL measuring cup, repeated 10 times per frequency group. The experiment was conducted using standard flat - fan nozzles. Spray nozzle models FPV110-015, FPV110-02, FPV110-03, and FPV110–04 were used, with spray pressures set to 0.2–0.35 MPa according to nozzle parameters. The experiment was repeated 10 times for averaging. Results confirmed the effects of rapid solenoid valve switching on flow regulation, providing a basis for determining the optimal operating speed of the variable-rate system and improving kale disease control efficiency in large fields.

## 3 Results and discussion

### 3.1 Variable rate spraying system identification model performance results and analysis

Performance testing of five models was conducted using a kale dataset from open-field cultivation. **Table 1** presents the performance test results of models incorporating different attention mechanisms. The data presented in the table reveals a notable similarity between the original YOLOV8 model’s detection accuracy and average image processing time and the results reported by (Kong et al., 2024). This correlation strongly suggests that the algorithm demonstrates a high degree of reliability in detecting kale. Nevertheless, within the specific parameters of this agricultural setting, the efficacy of the detection algorithm is but one critical component. Of equal importance is the average image processing time, which must be meticulously coordinated with the operational parameters of the variable spraying control system. The high plant density and partial occlusion in field kale cultivation pose challenges. Although YOLOv5s shows relatively high accuracy (86.3%) in testing, its performance in recognizing occluded kale decreases. This might be due to its limited adaptability to field conditions.

**Table 1:** Comparison of mainstream model performance test result.

Model	Accuracy (%)	Recall (%)	mAP@0.5 (%)	Average image Processing Time (ms)
YOLOv8	88.1	77.9	87.9	22.7
SSD	81.9	72.4	81.7	29.3
Faster R-CNN	91.5	82.1	91.3	135.6
YOLOv5s	86.3	80.3	86.2	25.6
YOLOv8n	88.6	78.1	88.7	20.3

**Figure 6:**
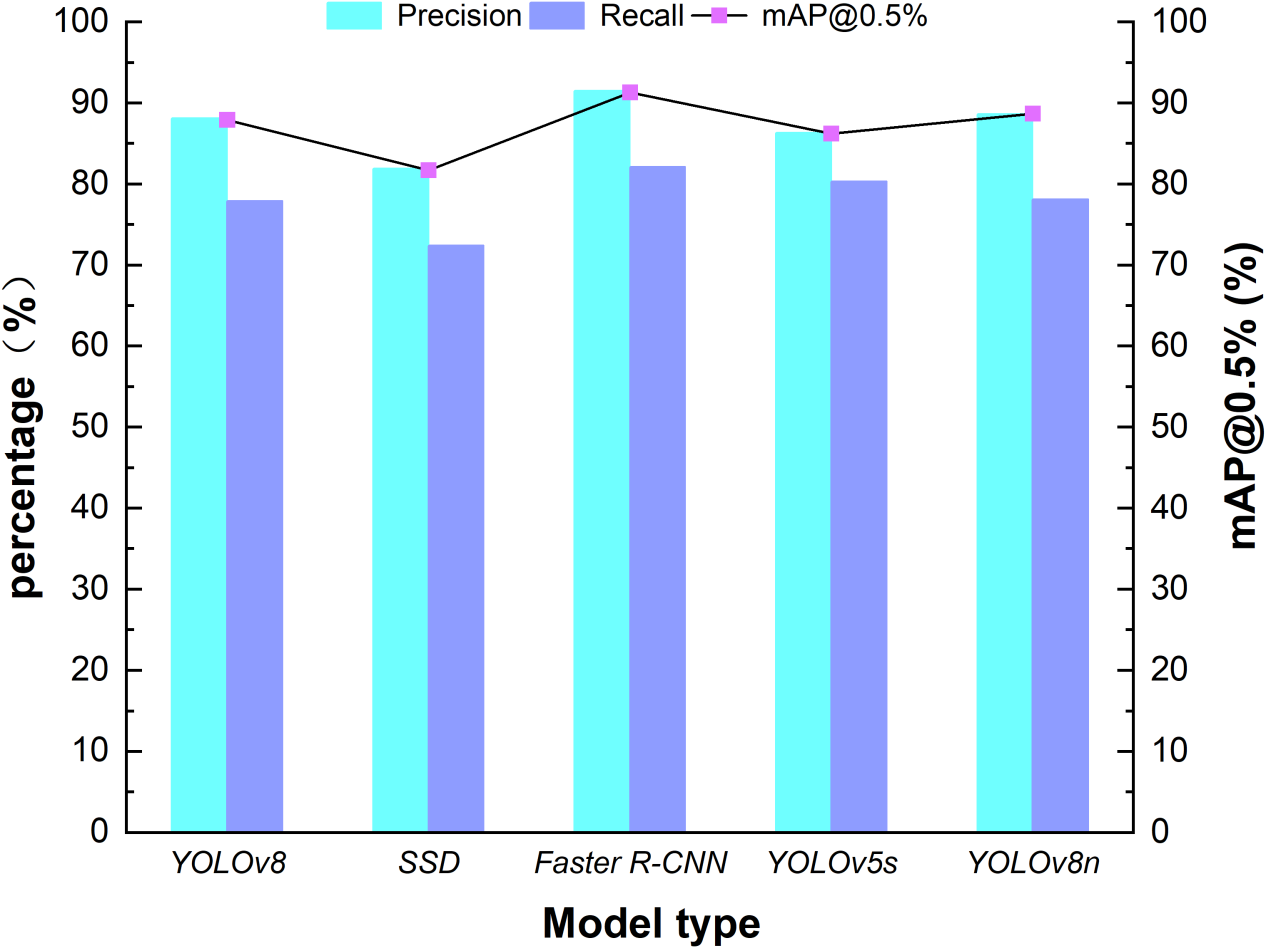
Performance test results of different models.

Following the comparison test of model performance, **Figure 6** shows the performance comparison of the models with various attention mechanisms. Although Faster R-CNN demonstrates a certain advantage in detection accuracy, reaching up to 91.5%, its average image processing time exceeds 135.5 ms, posing a challenge for real-time detection in high-density field kale environments. In contrast, the improved YOLOv8n model achieved a mAP@0.5 of 88.6 ± 0.3%, which is 0.5% higher than the original model. Its average single-frame image processing time is 20.3 ± 0.5 ms—accelerated by 0.05%—meeting the demands of real-time field detection. Moreover, the refined model outperforms the original one in image processing speed, making it more suitable for real - time field applications. This enhanced efficiency, coupled with its superior adaptability to high - density planting conditions, provides robust technological support for precise pesticide application. The results show that YOLOv8n can accurately recognize kale in high-density conditions, providing reliable feedback for the variable rate spraying system. The recognition accuracy, with a reliability rate of 88.6%, reduces missed detections and ensures effective pest management.

### 3.2 Test results and analysis of practical field application of the target center canopy identification model

To validate the effectiveness of the improved YOLOv8n model in actual field detection tasks, a comparison with the original YOLOv8 benchmark model was conducted on a large-field kale test dataset (N = 1783, with masked samples), as shown in **Table 2**. The results show that the average mAP@0.5 improved to 88.7%, an increase of 0.8% over the original model, indicating enhanced feature extraction capability for small and occluded targets. The YOLOv8n Kale Keypoints model improved by Zheng et al. (2024) mainly focuses on detecting kale heads, achieving an average precision (AP50-95) of 99.2%, but with a relatively large error. In contrast, this study ensures more stable real - time kale field detection with smaller errors. The single-frame processing time was reduced to 20.3 ± 0.5 ms, and inference speed (FPS) improved by 10.74%, meeting the requirements of real-time crop detection. Based on the detection results, the variable rate spraying system implements a partitioned execution strategy. Each detection region corresponds to a nozzle, and when the predicted box intersects with the execution box beyond a set threshold, the upper computer sends spraying commands via serial communication. The lower computer then calculates the dynamic delay based on machine speed to control solenoid valve timing (Silva et al., 2018).

**Table 2:** Comparison of improvement results of field kale identification model of YOLOv8.

Model	mAp@0.5 (%)	Flops (G)	Fps (s)	Params (M)	Per-Frame Inference Time (ms)
YOLOV8	87.9	10.6	76.3	7.5	22.7 ± 0.8
YOLOV8n	88.7	9.1	84.5	6.8	20.3 ± 0.5

**Figure 7:**
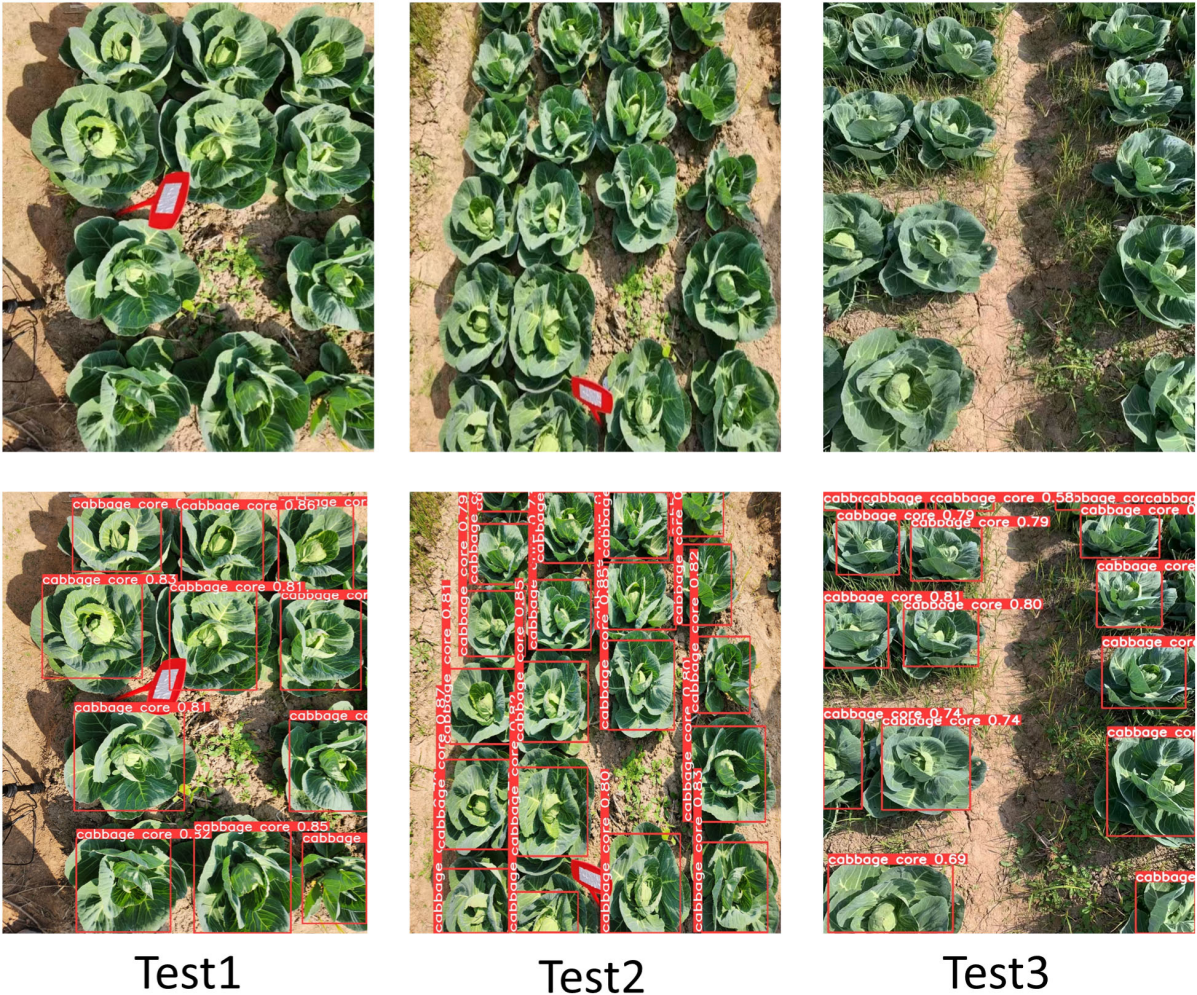
Improvement of YOLOv8n detection of kale in real fields.

**Figure 8:**
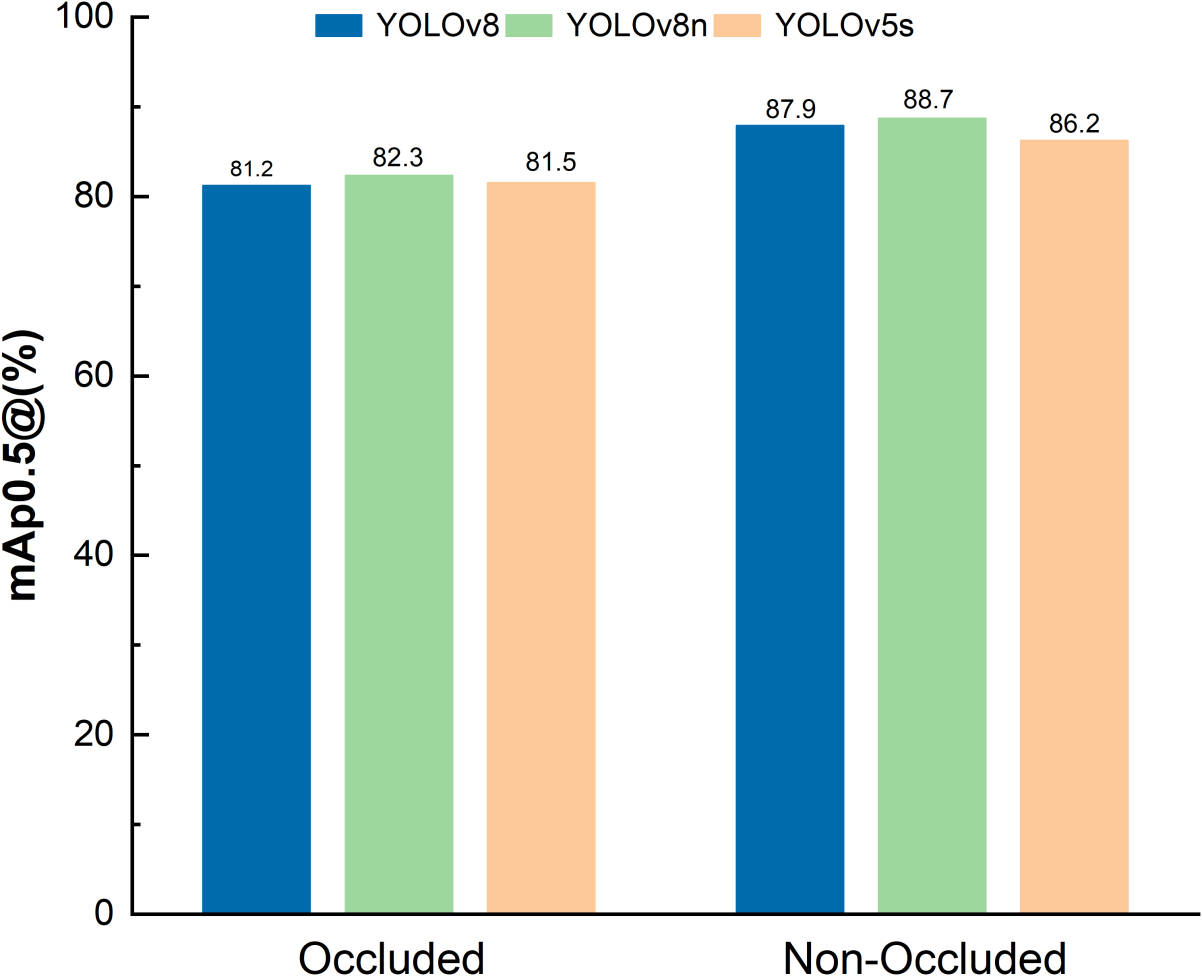
Comparative accuracy test results of the three models on top of the dataset.

Validation conducted in Yueyang City, Hunan Province, showed significant improvements in detection performance. The improved YOLOv8n model achieved 88.7% recognition accuracy in high-density kale fields (plant density: 5.2 plants/m²), with a low repeat detection and leakage rate, as shown in **Figure 7**. Under adequate lighting, the model maintained a low repeat detection rate, and in scenes with partial occlusion and high density, the missed detection rate was below 11.3%.The actual test results are close to those of Ong et al., who used CNN for in - field kale weed detection (Ong et al., 2023). The improved model could be tested in future applications of precise pesticide spraying on kale fields with more weeds to evaluate its real - world effectiveness. In addition, under optimal conditions—no occlusion from weeds or field signage—the recognition accuracy reached 88.7%. This demonstrates that the improved YOLOv8n is significantly more robust and adaptable in complex field environments, and capable of precise operation even under occlusion, as shown in **Figure 8**.

### 3.3 Results and analysis of PWM duty cycle-flow spray modeling

#### 3.3.1 Spray flow accuracy test results and analysis

**Figure 9:**
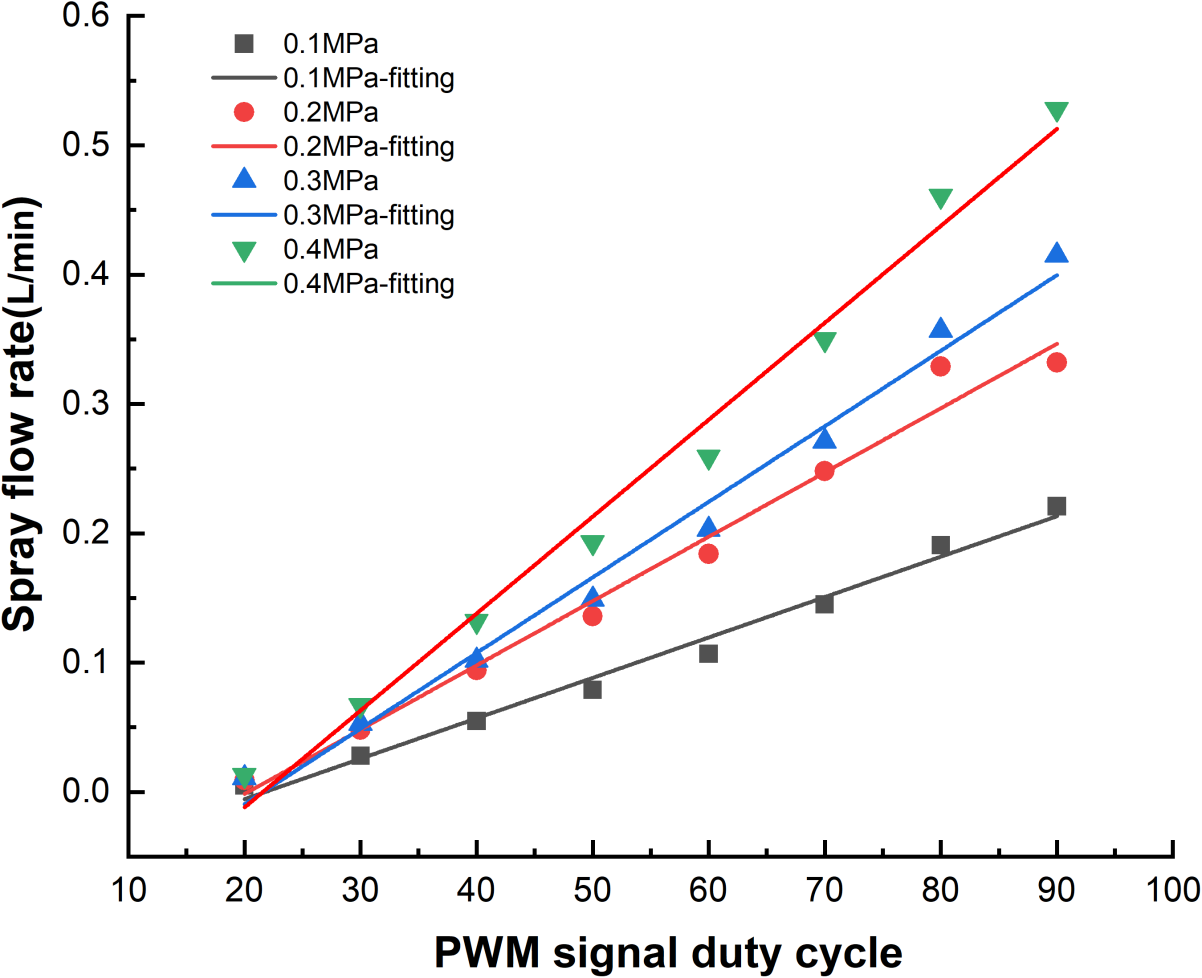
Flow spray fitting results for solenoid valves with different duty cycle signals.

A systematic experimental design was used to analyze the relationship between PWM duty cycle and spray flow under fan-shaped anti-drift nozzles at pressure levels from 0.1–0.4 MPa. Flow test data were processed via linear regression analysis using Origin 2021. The results of solenoid valve flow response to different duty cycle signals are shown in **Figure 9**, based on the average of five test groups. The theoretical and actual flow rates closely matched. The regression coefficients (R^2^) for the spraying models under four pressures were all above 0.9, indicating strong linearity. Additionally, in similar studies, Han et al. (2024) achieved significant success using an energy - saving PWM - based electromagnetic - valve control approach. This underscores the effectiveness of PWM methods in delivering precise electromagnetic - valve control. The composite model average was R² = 0.9958, meaning the PWM duty cycle signal explained 99.58% of the flow rate variation. The maximum relative error was 4.1%, meeting the ISO 5628-1:2017 Plant Protection Machinery-Spraying Equipment technical standard for precision spraying operations. This error threshold is below 5%, aligning with the requirements for accurate application in high-density crops like kale. As system pressure increased from 0.1 MPa to 0.4 MPa, flow rates also increased, consistent with fluid dynamics theory. When the duty cycle was below 20%, the flow dropped below 0.01 L/min due to solenoid valve mechanical delay and the minimum pressure threshold. In the 40%–80% duty cycle range, the coefficient of variation (CV) was less than 2%, confirming suitability for variable spraying applications, with minimal influence from signal changes.

#### 3.3.2 Spray canopy flow accuracy test results and analysis

**Figure 10:**
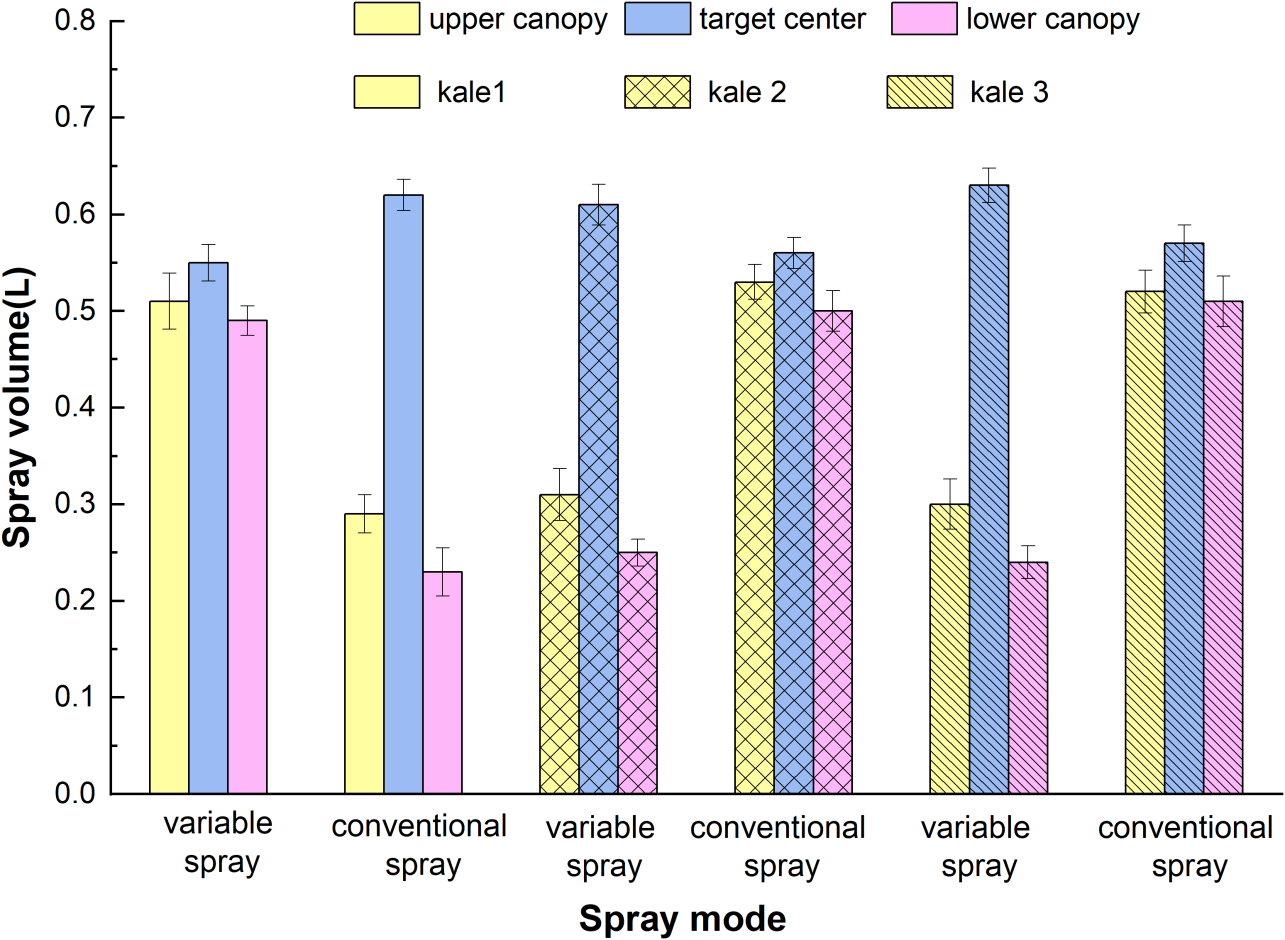
Test results of spray volume trials in different canopy areas.

In the performance test of kale spraying across different canopy areas, spray volume per test kale and canopy area were recorded. Results are shown in **Table 3**, comparing continuous and variable spraying. The average spray volume under variable spraying in three replicated groups was significantly higher than under continuous spraying, and the CV decreased by 0.2%. For kale canopy edge zones, spray volume was reduced by 42.31% at the upper edge and 52% at the lower edge, significantly improving pesticide targeting at the bullseye. However, environmental airflow caused some spray drift, and leaf interference affected droplet penetration. Overall, the variable-rate spraying system demonstrated clear advantages over constant spraying in targeting kale pest control. The results showed that spray distribution was significantly improved under variable spraying. As seen in **Figure 10**, the spray volume in the bullseye region increased by an average of 10.7% compared to constant spraying, closely aligning with control requirements for pest management (Seol et al., 2022). The similar variable and constant spraying results in different kale groups may stem from spray drift during the test (Li et al., 2021). This has minimal impact on overall pest control in extensive kale fields.

**Table 3:** Comparison of spraying effects between continuous and variable rate spraying.

Spraying method	Upper canopy edge			Target center			Lower canopy edge		
	Test no.	Spray volume (L)	Average (L)	Test no.	Spray volume (L)	Average (L)	Test no.	Spray volume (L)	Average (L)
Constant rate spraying	01	0.51	0.52	01	0.55	0.56	01	0.49	0.50
	02	0.53		02	0.56		02	0.50	
	03	0.52		03	0.57		03	0.51	
Variable rate spraying	01	0.29	0.30	01	0.62	0.62	01	0.23	0.24
	02	0.31		02	0.61		02	0.25	
	03	0.30		03	0.63		03	0.24	

### 3.4 Field test results and analysis

#### 3.4.1 Verification test results and analysis of atomized deposition performance

During field tests, droplet deposition data from different spraying methods were obtained via DepositScan software analysis of water-sensitive paper. As per NY/T 650–2013 agricultural standards for kale pest control, spray deposition density should exceed 85 drops/cm², and coverage should surpass 20%. As shown in **Table 4**, compared to constant spraying, the CV-based variable-rate method achieved a droplet deposition density increase of 28.45% and deposition volume increase of 0.87 µL/cm^2^—an improvement, though not drastic. These results generally meet on-target spraying standards. In the constant mode, coverage was 22.93%, with some fluctuation likely due to spray drift, which caused non-target deposition and droplet coalescence, reducing the number of distinct droplets. The reason for these results is similar to Zhang’s findings on PWM - based variable spraying (Zhang C. et al., 2024). This further shows that spray drift can affect the system’s deposition performance. In the CV-based variable-rate mode, the rapid response of the vision module ensured accurate flow control. The solenoid valve, controlled via varying PWM duty cycles, switched frequently and generated a water hammer effect (Liu et al., 2014), producing smaller droplets. Consequently, droplet count increased in variable spraying, with a variation of 0.33 µL/cm^2^.

**Table 4:** Deposition parameters under different spraying modes.

Spraying mode	Flat-fan nozzle		
	Deposition density (droplets/cm^2^)	Deposition amount (uL/cm^2^)	Coverage (%)
Constant-rate	95.92	0.54	22.93
CV-based variate-rate	115.74	0.87	28.45

#### 3.4.2 Droplet deposition test results and analysis

**Figure 11:**
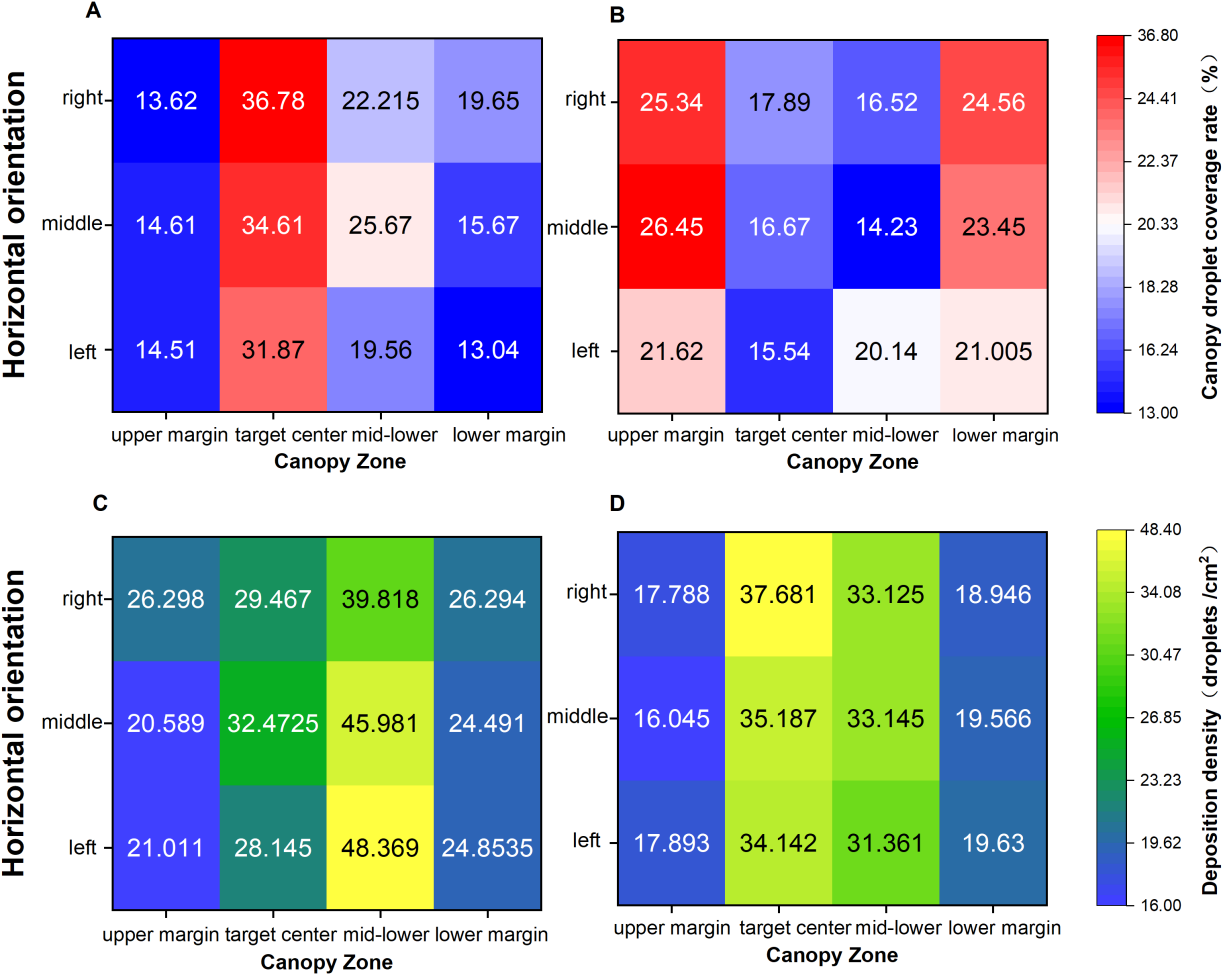
Test results in different spraying modes: **(A)** Variable-mode droplet coverage **(B)** Constant-mode droplet coverage **(C)** Constant-mode deposition Density **(D)** Variable-mode density.

At the end of the test, atomized deposition on water-sensitive paper was scanned using the Aficio MP 7502 scanner (Deep Xiangyu Technology Co., Ltd., Ricoh Group, RICOH) at a resolution of 600 × 600 ppi. DepositScan was used to process the grayscale images and extract droplet coverage and deposition density to evaluate the spray effect test indices. Based on the droplet deposition distribution data, Origin 2021 software was used to generate distribution maps of the mean droplet coverage and deposition density across the kale canopy area in the field. The droplet deposition density under different spray modes is shown in **Figures 11A, B**. Compared with constant spraying, the deposition density in the canopy target center under variable-rate spraying was significantly higher, with an average increase of 16.1%, indicating that the variable-rate system better achieves targeted spraying. This contributes to improved pest and disease control in the plant core. In contrast, constant spraying resulted in high deposition in canopy edge areas (non-target zones), with wide-area coverage leading to pharmaceutical waste. In both modes, droplet deposition density exceeded 16 drops/cm^2^, meeting GB/T 8231, Guidelines for the Rational Use of Pesticides in China. Furthermore, the significantly higher deposition density in the target center under variable-rate spraying is attributed to the binocular vision sensor’s rapid target acquisition and the solenoid valve’s frequent switching, which increases local nozzle pressure. The system can thus adjust the flow rate according to variations in the canopy volume grid at different locations, enabling precise application. Regarding droplet coverage under both modes (**Figures 11C, D**), results from the variable spraying test show that average droplet coverage at the target center reached 34.42%, representing a 17.72% increase compared to constant spraying. Although the water hammer effect from frequent solenoid valve switching may affect results, variable-rate spraying still showed good droplet uniformity and stronger penetration.

#### 3.4.3 Solenoid valve dynamic response and variable spraying performance validation results and analysis

**Figure 12:**
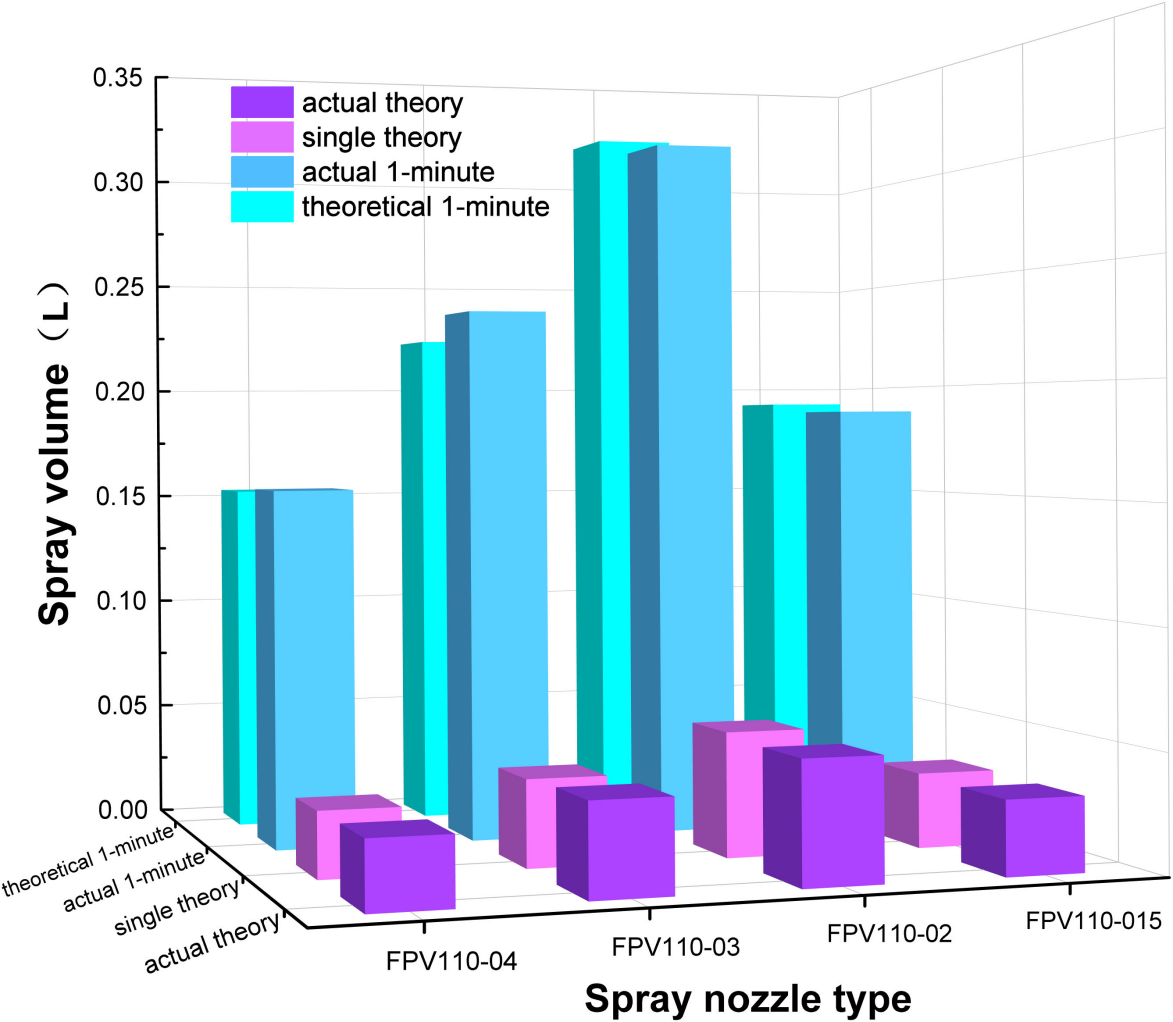
Comparison of theoretical and actual spraying volume results.

Testing the system’s performance at different travel speeds revealed that at speeds over 2 m/s, the binocular vision sensor could still identify kale targets; however, the solenoid valve’s frequent switching hindered accurate measurement of spray volume per pass. Therefore, in this test, the valve’s opening time was set to 0.5 s and closing time to 1 s to ensure accurate measurement at 2 m/s. To minimize error, each test was repeated five times under the same conditions, and the average values were taken, as shown in **Figure 12**. As indicated in the figure, the system’s theoretical spray volume aligns closely with the actual spray volume. Here, “theoretical” refers to the system’s theoretical flow rate, while “actual” denotes the measured value. This indicates the electromagnetic valve’s flow reliability is effective. The corresponding single and cumulative spray volumes for four travel speeds over 1 min are shown in **Table 5**. Results show that increased speed significantly impacts spray volume. At 2 m/s, the FPV110–04 nozzle showed a single-pass error of 1.79%, while the FPV110–03 at 1.5 m/s had an error of 4.64%, indicating a strong influence of travel speed on actual volume. The influence of pressure on spray volume aligns with the findings of Dai et al. (2019) on electromagnetic valve - controlled spray flow characteristics under dynamic conditions. Thus, it’s essential to conduct tests on the actual flow values of variable - rate spraying systems. Higher spray pressure increased atomization, enabling droplets to better penetrate dense canopies at high speeds and reducing under-coverage. However, at low pressure (e.g., FPV110–015 at 0.20 MPa), fog droplets lacked kinetic energy and were prone to rebounding, contaminating non-target areas and diverging from theoretical values. This suggests that the system performs best at a medium travel speed of 1 m/s for effective kale pest and disease control.

**Table 5:** Spray volume acquisition results of variable rate spraying control system.

Spray nozzle model	Spray pressure (MPa)	Travel speed (m/s)	Duty cycle (%)	Actual spray volume (L)		Theoretical spray volume (L)		Relative error (%)	
				Single	1-Minute	Single	1-Minute	Single	1-Minute
FPV110-015	0.20	0.5	50	0.0313	0.188	0.0320	0.180	4.15	4.26
FPV110-02	0.25	1	50	0.0513	0.308	0.0530	0.318	3.31	3.25
FPV110-03	0.30	1.5	50	0.0388	0.233	0.0370	0.222	4.64	4.72
FPV110-04	0.35	2	50	0.0285	0.155	0.0280	0.153	1.79	1.31

## 4 Conclusions

This study developed a precise variable-rate spraying system for high-density kale fields by integrating RealSense binocular vision with PWM-based variable-rate control technology, achieving efficient precise spraying and pest management. Field trials confirmed the effectiveness of the relevant models. The main findings and conclusions are as follows:

1. The improved YOLOv8n algorithm greatly boosted detection speed and accuracy. It handles high - density situations and real - time precise operations. Its single - frame image processing time is 20.3 ms, 10.7% faster than the original model. With a detection accuracy of 88.6%, it exhibits strong robustness in complex scenarios.
2. A target - center canopy spray model was built based on kale canopy morphology. It enhanced deposition at the target center and cut pesticide use, aligning with precision - application standards. The model showed a strong correlation between theoretical and actual flow rates (R² = 0.9958), greatly improving pesticide - saving rates.
3. Field testing of the RealSense D455 - based variable - rate spraying system showed it considerably lowered deposition in non - sprayed areas. With good target - spraying performance, it achieved an average coverage of 34.42% and reduced pesticide use by 26.58%. The system performed optimally at 1 m/s, meeting precision - application needs.

In summary, field trials confirmed that the CV-based variable-rate spraying system significantly improved target area droplet deposition, increased pesticide utilization by 26.58%, and reduced drift to non-target zones. These results align with standard application practices and enhance kale pest and disease control. However, limitations remain. The study did not address system adaptation to non-standard agronomic conditions or quantify the influence of weeds on detection accuracy. The long-term stability of the detection model under dynamic canopy changes also requires further research. Future work will focus on canopy growth modeling and edge computing optimization to achieve full-cycle precision control in complex field environments. This will help reduce pesticide waste and improve kale pest management outcomes.

## Data Availability

The original contributions presented in the study are included in the article/**Supplementary Material**. Further inquiries can be directed to the corresponding author.
