# Supplementary material for: Design and testing of a RealSense-based variable spraying control system for field kale
Source: Front Plant Sci. 2025 Aug 4;16:1618159. doi: 10.3389/fpls.2025.1618159 (PMC12358357; doi:10.3389/fpls.2025.1618159)

Supplementary Material

# Supplementary Figures and Tables

## Supplementary Figures

**Supplementary Figure 1(A).** Basic components of the system


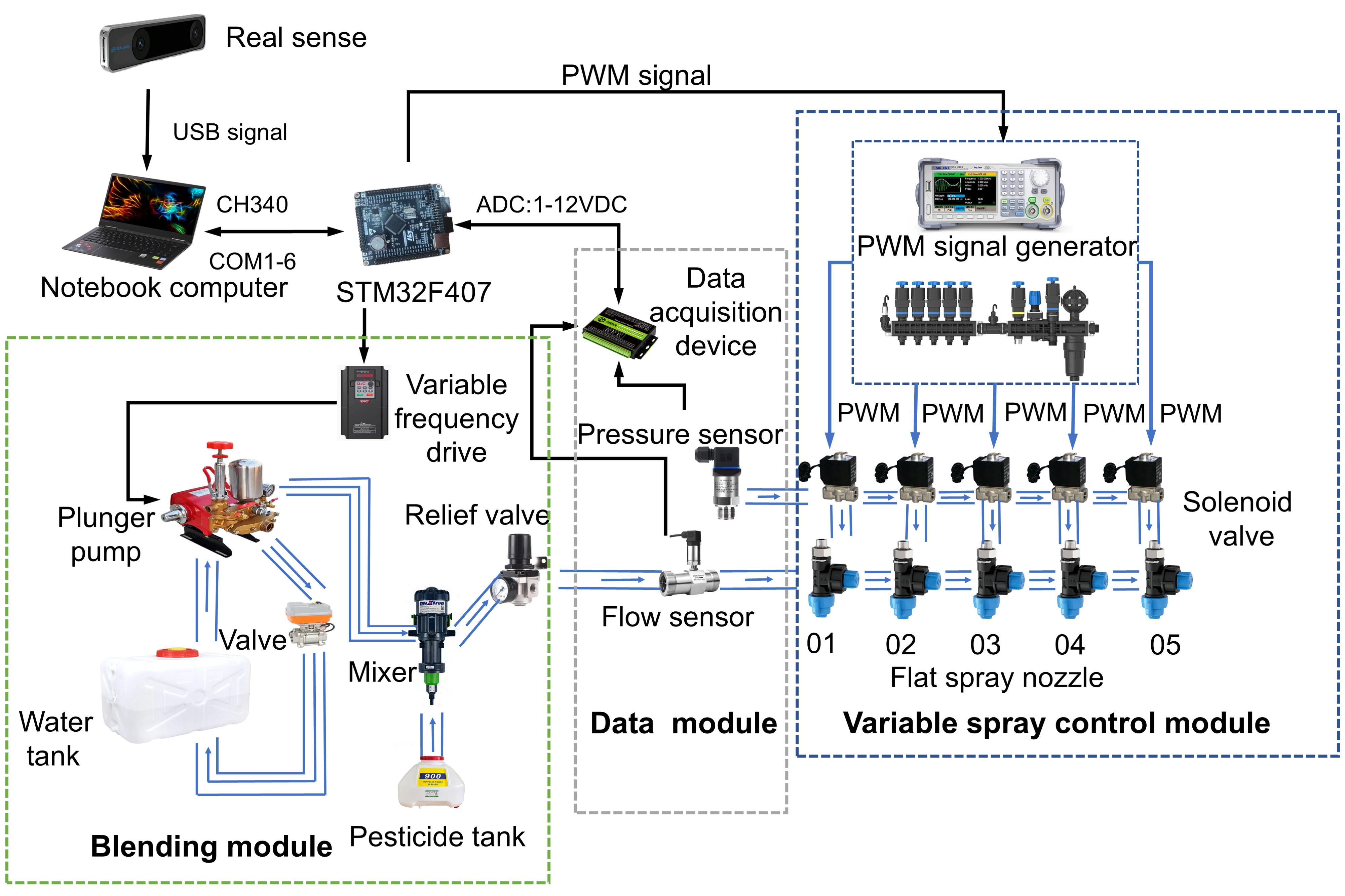


**Supplementary Figure 1(B).** Overall structure


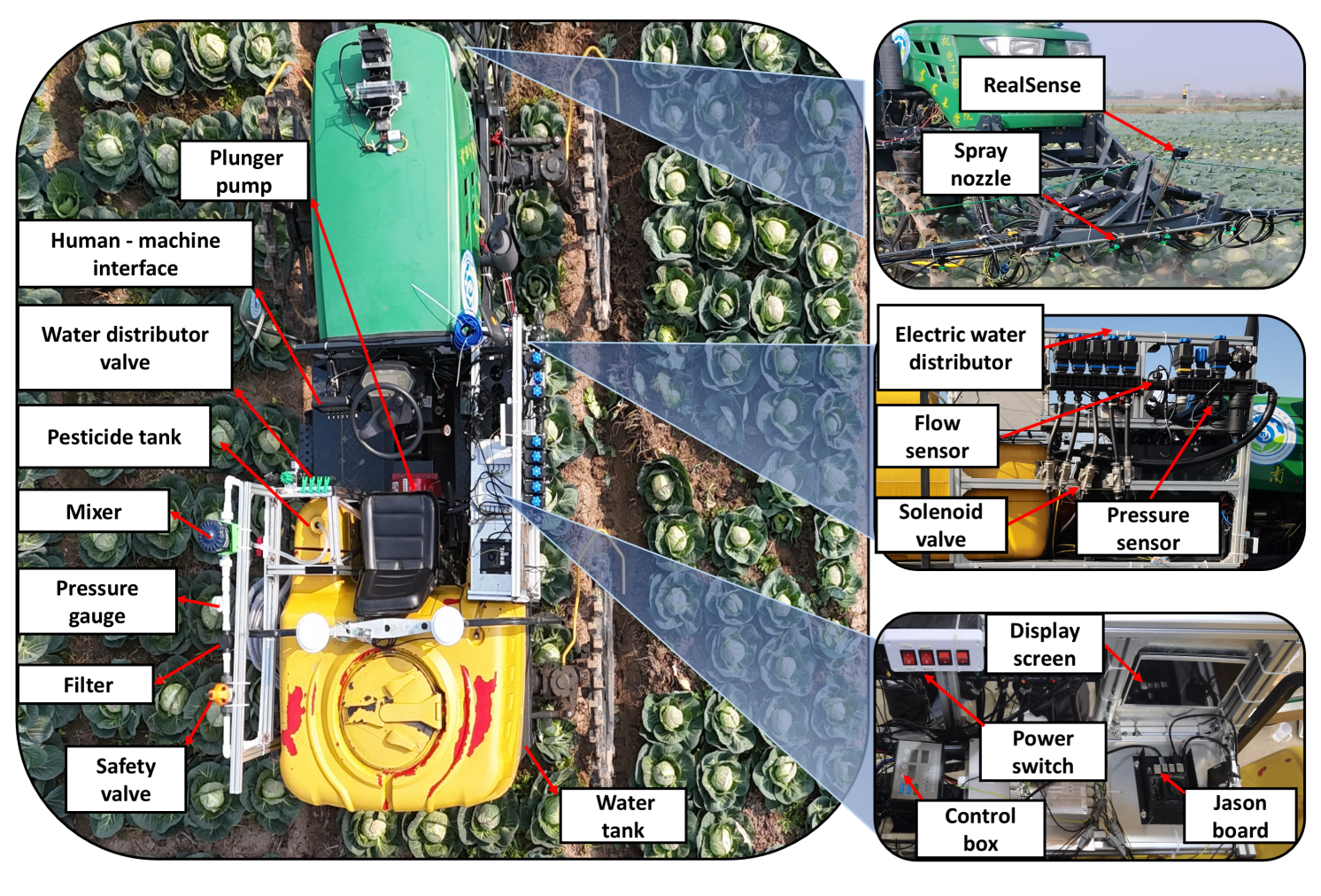


**Supplementary Figure 5（A）.** Water-sensitive paper sampling point layout


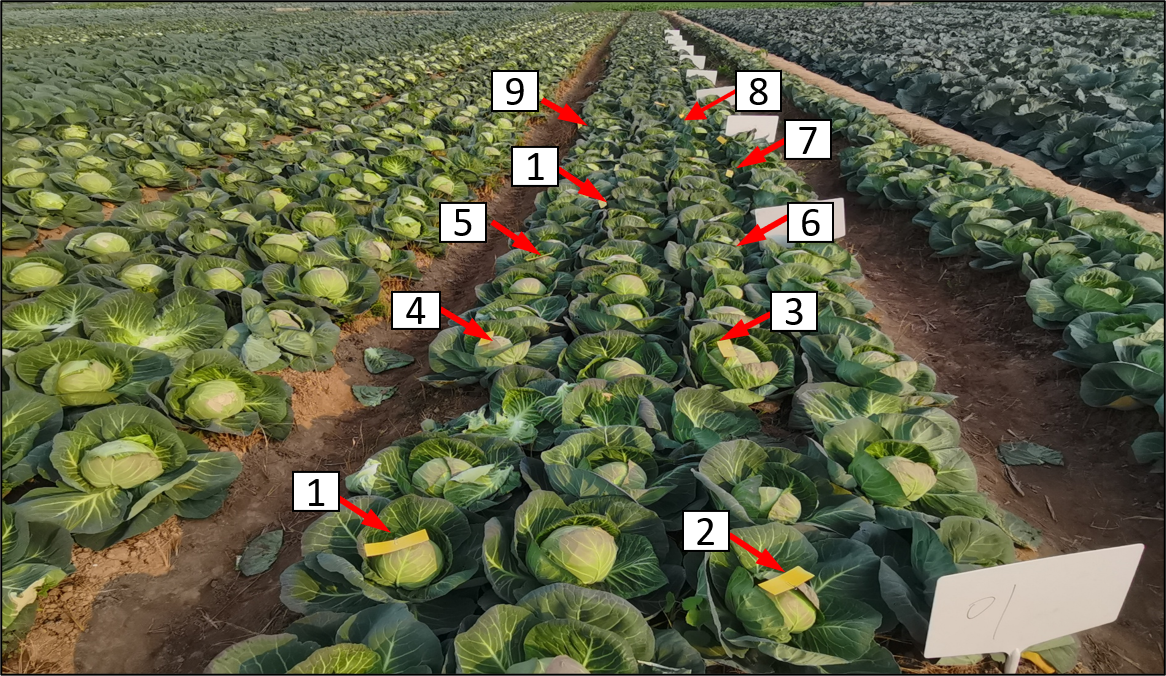


**Supplementary Figure 5（B）.** Schematic layout of atomized deposition


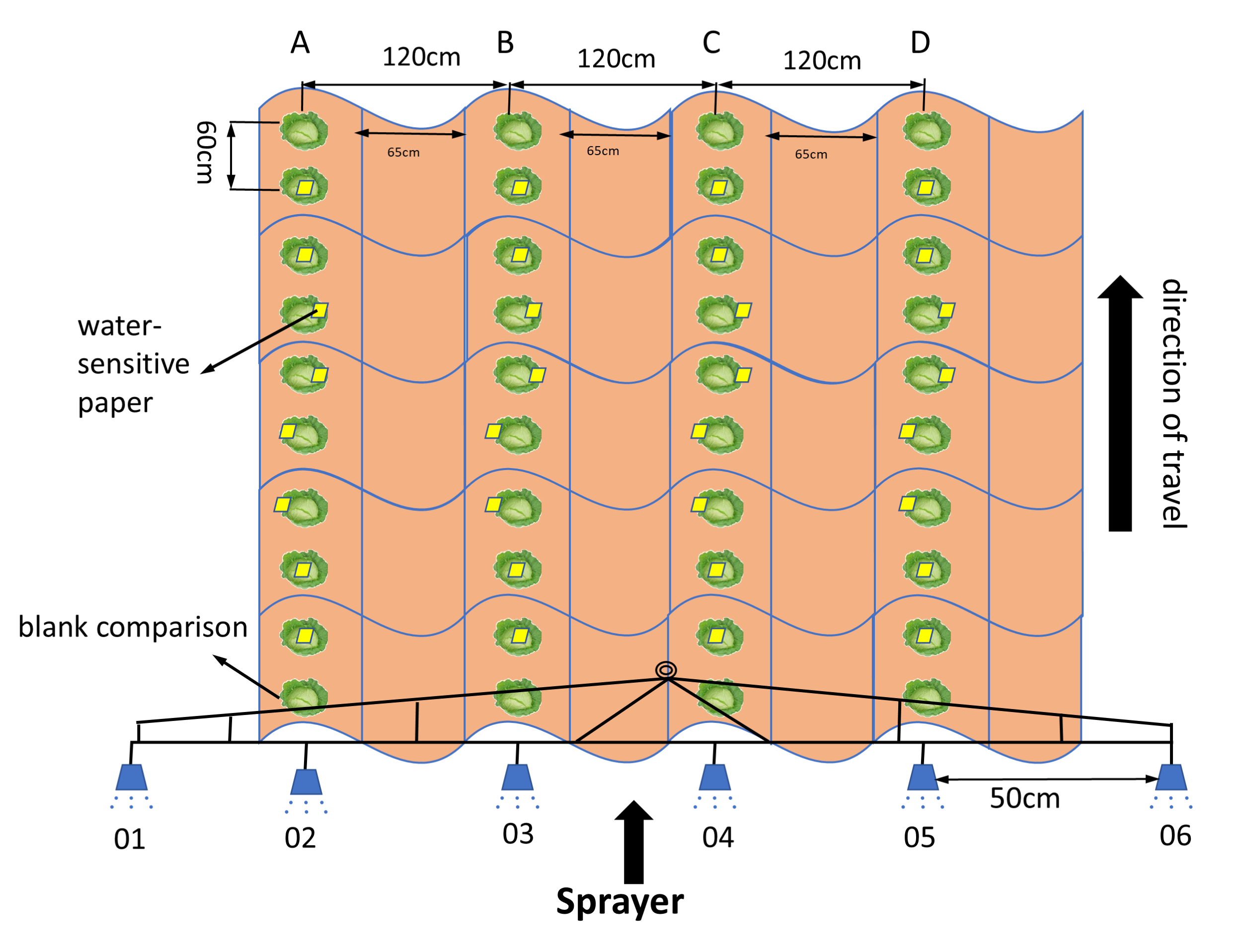


**Supplementary Figure 5(C).** Water-sensitive paper after field experiment tests


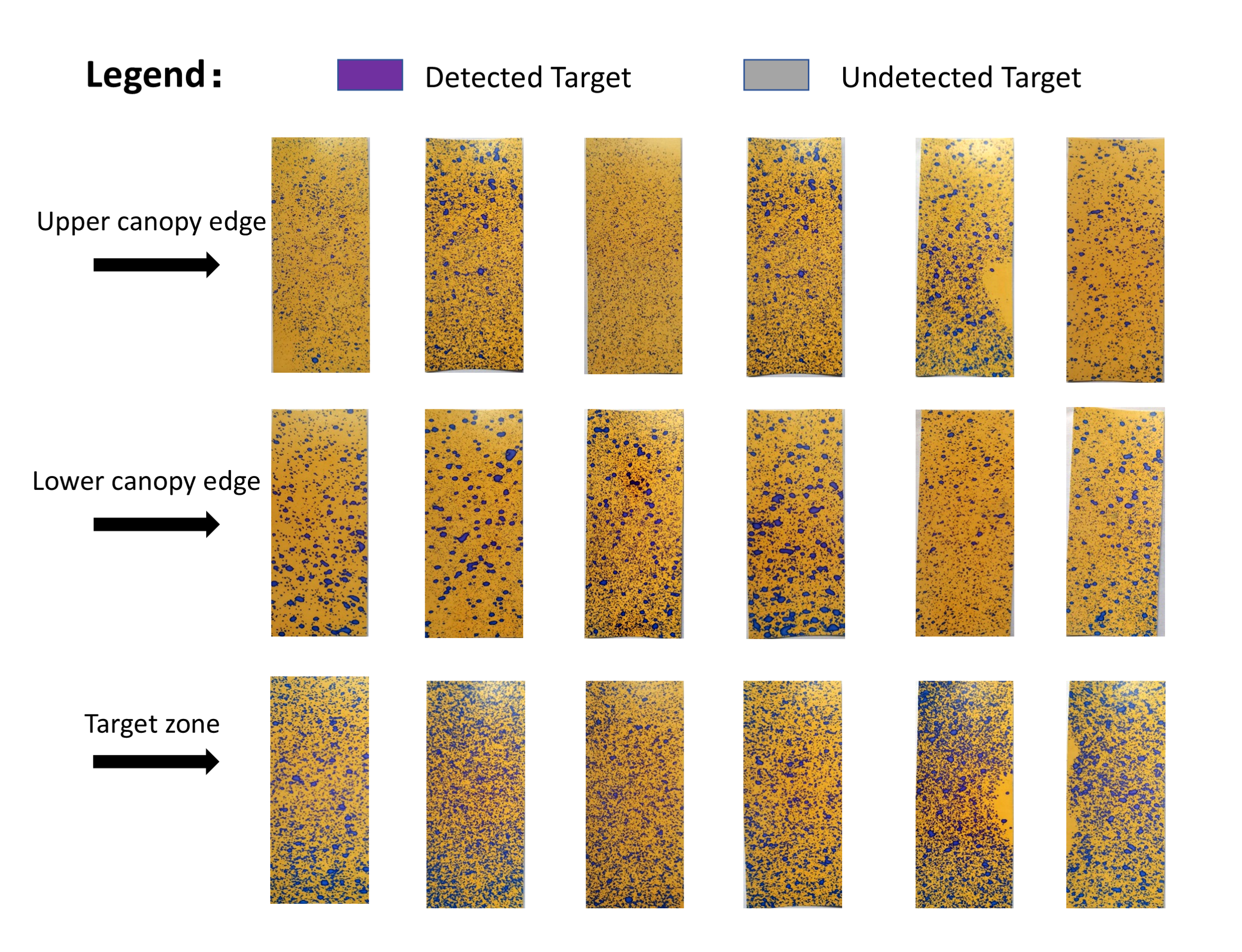


**Supplementary Figure 5（D）.** Field test experiment site


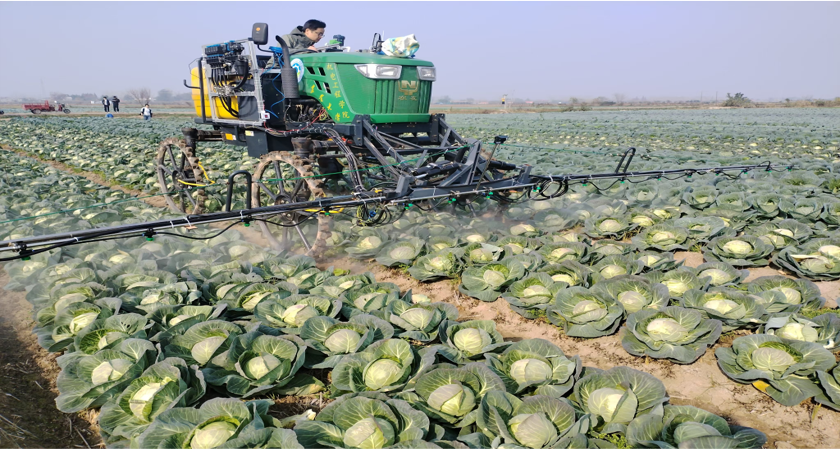


**Supplementary Figure 11(A).** Variable-mode droplet coverage


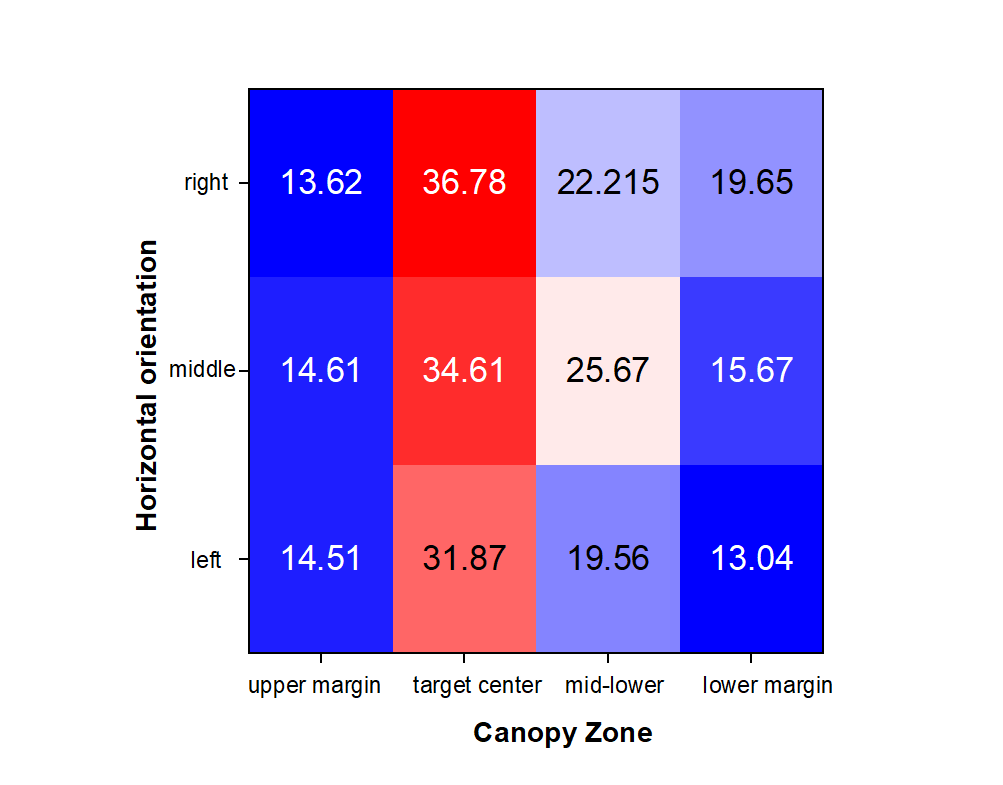


**Supplementary Figure 11(B).** Constant-mode droplet coverage


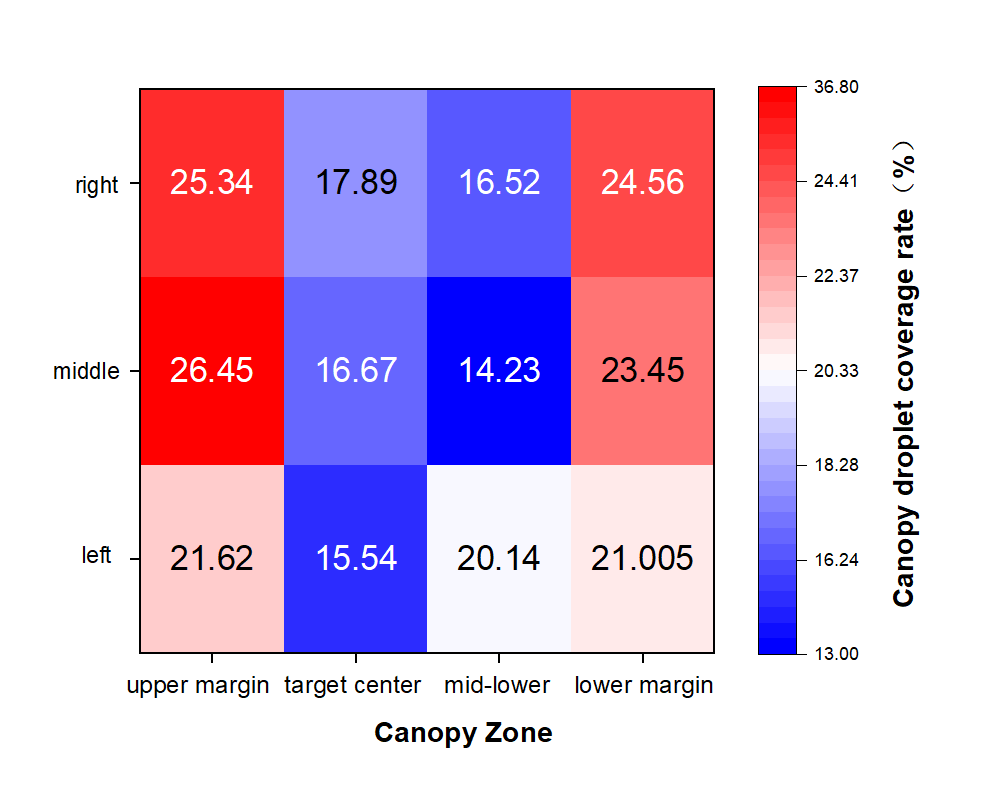


**Supplementary Figure 11(C).** Constant-mode deposition Density


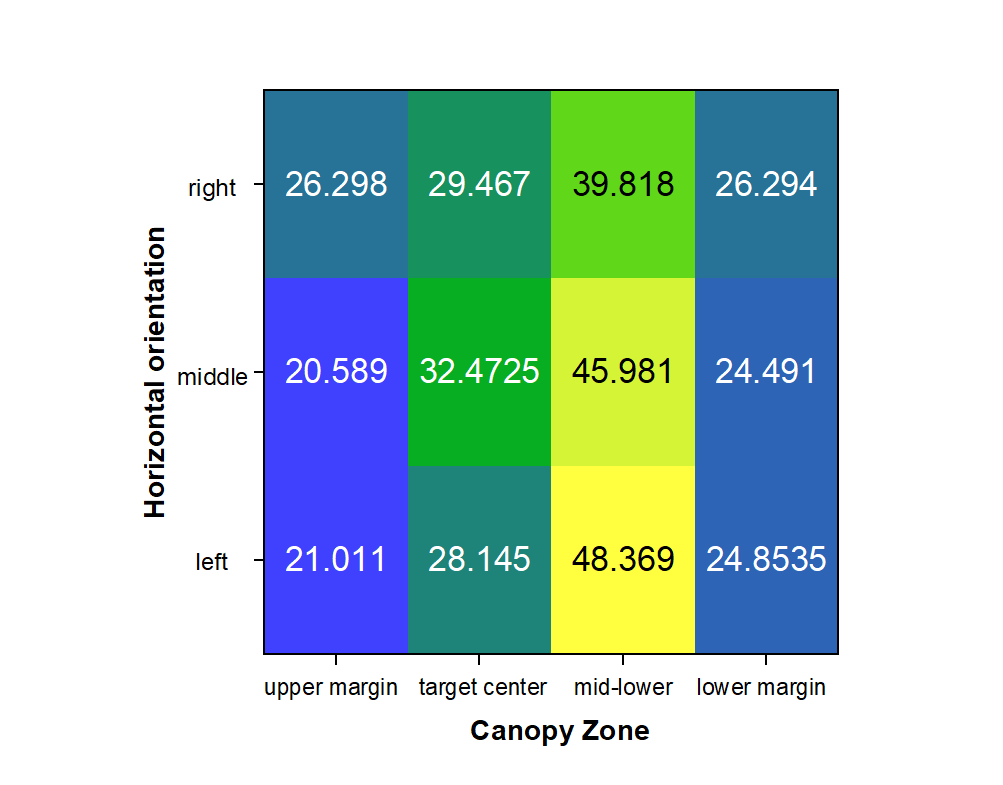


**Supplementary Figure 11(D).** Variable-mode density


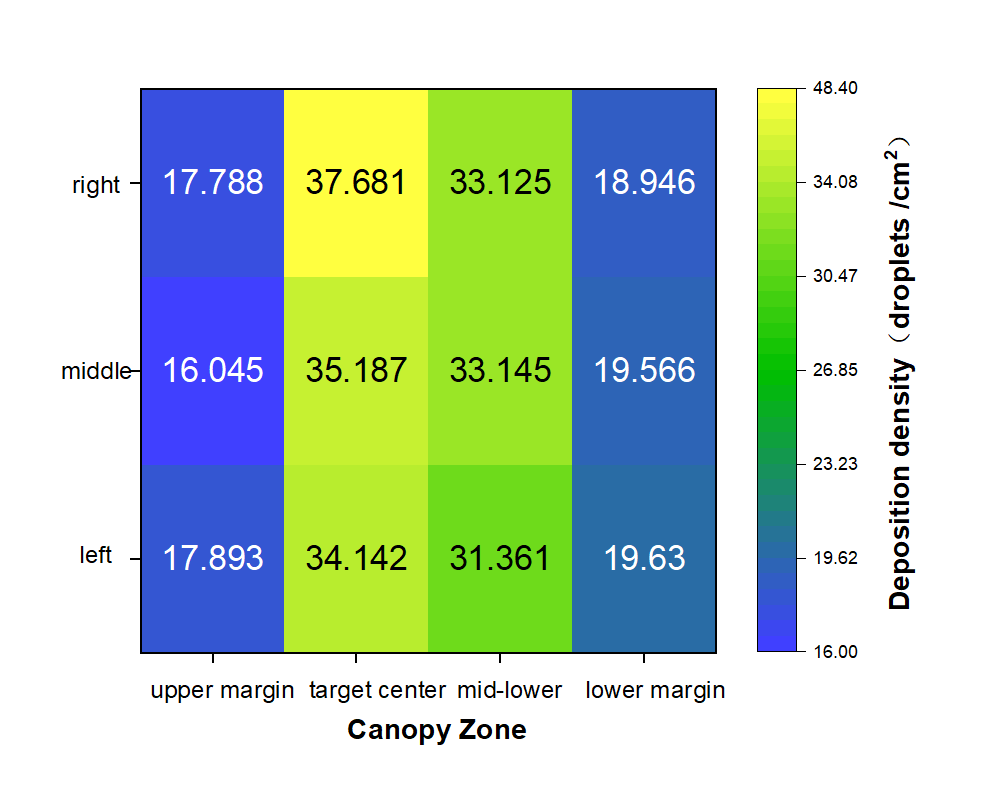

Supplement: Supplementary file 1 [file DataSheet1.docx]
